# Regional and global shifts in crop diversity through the Anthropocene

**DOI:** 10.1371/journal.pone.0209788

**Published:** 2019-02-06

**Authors:** Adam R. Martin, Marc W. Cadotte, Marney E. Isaac, Rubén Milla, Denis Vile, Cyrille Violle

**Affiliations:** 1 Department of Physical and Environmental Sciences and The Centre for Critical Development Studies, University of Toronto Scarborough, Scarborough, Canada; 2 Department of Biological Sciences, University of Toronto Scarborough, Scarborough, Canada; 3 Departamento de Biología y Geología, Física y Química Inorgánica, Universidad Rey Juan Carlos, Móstoles, Spain; 4 Laboratoire d’Ecophysiologie des Plantes sous Stress Environnementaux (LEPSE, UMR759), Institut National de la Recherche Agronomique (INRA), Université de Montpellier, Montpellier, France; 5 UMR 5175, Centre d’Ecologie Fonctionnelle et Evolutive, Université de Montpellier, Université Paul Valéry, EPHE, Montpellier, France; Instituto Agricultura Sostenible, SPAIN

## Abstract

The Anthropocene epoch is partly defined by anthropogenic spread of crops beyond their centres of origin. At global scales, evidence indicates that species-level taxonomic diversity of crops being cultivated on large-scale agricultural lands has increased linearly over the past 50 years. Yet environmental and socio-economic differences support expectations that temporal changes in crop diversity vary across regions. Ecological theory also suggests that changes in crop taxonomic diversity may not necessarily reflect changes in the evolutionary diversity of crops. We used data from the Food and Agricultural Organization (FAO) of the United Nations to assess changes in crop taxonomic- and phylogenetic diversity across 22 subcontinental-scale regions from 1961–2014. We document certain broad consistencies across nearly all regions: i) little change in crop diversity from 1961 through to the late 1970s; followed by ii) a 10-year period of sharp diversification through the early 1980s; followed by iii) a “levelling-off” of crop diversification beginning in the early 1990s. However, the specific onset and duration of these distinct periods differs significantly across regions and are unrelated to agricultural expansion, indicating that unique policy or environmental conditions influence the crops being grown within a given region. Additionally, while the 1970s and 1980s are defined by region-scale increases in crop diversity this period marks the increasing dominance of a small number of crop species and lineages; a trend resulting in detectable increases in the similarity of crops being grown across regions. Broad similarities in the species-level taxonomic and phylogenetic diversity of crops being grown across regions, primarily at large industrial scales captured by FAO data, represent a unique feature of the Anthropocene epoch. Yet nuanced asymmetries in regional-scale trends suggest that environmental and socio-economic factors play a key role in shaping observed macro-ecological changes in the plant diversity on agricultural lands.

## Introduction

A major line of scientific evidence defining the Anthropocene epoch–the period of Earth’s history defined by the dominance of humans–is the human-caused changes in Earth’s biodiversity and biogeography. While there are multiple dimensions to changes in biogeography in the Anthropocene [1], changes in the human-mediated spread of crops beyond their regions of domestication into other parts of the world are central to these arguments [2, 3]. Some of the most prominent shifts in crop biogeography occurred during the “Columbian Exchange”: a major interchange of commercially important plant species during in the 15^th^ and 16^th^ centuries between the Old and New Worlds [4–6]. Yet more recently since the 1950s, global-scale analyses indicate that there have been major influxes (and subsequent domination) of crops into the food supplies, diets, agricultural economies, and farmlands in many parts of the world [3, 7, 8].

Global-scale assessments of the changes in crop diversity over recent decades have largely reported linear increases in the crop diversity (and evenness) associated with global diets and agricultural economies, with these patterns being key for understanding trends in food production, consumption, and trade [3, 7–9]. Yet while these patterns are certainly notable cf. Fig 2 in [7], there is reason to expect that the timing and rate of change in the diversity profiles of croplands has also differed drastically among regions. Specifically, agricultural trade liberalization and structural adjustment programs throughout the 1980s incentivized the production and export of a few select crops or genotypes, with major impacts on crop selection and management at the regional- or country-level e.g. [10, 11, 12]. Such programs overwhelmingly impacted developing regions, leading to the expectation that patterns of crop diversification over the past 50 years have likely differed drastically among regions. At the same time, climatic limitations to growing certain crops in higher latitudes also has likely led to less drastic or immediate shifts in agricultural diversity in these regions, as compared to lower latitudes e.g. [13]. To date, however, patterns of crop diversification have not been evaluated through regional comparisons of crop diversity change through time.

Furthermore, existing global evaluations of crop diversity have primarily focused on taxonomic diversity–measured as crop species richness–across global scales [3, 7]. Yet these analyses may overlook shifts in the evolutionary profiles of croplands. Ecological theory and research suggests that crop evolutionary diversity–referred to in the ecological literature and hereafter as “phylogenetic” diversity–would provide more mechanistic insights and predictions into the sustainability and functioning of agricultural systems, including net primary production, and resistance or resilience of crops to pests, pathogens, or environmental change [14–16]. However to date there remain no analyses evaluating if changing composition of agricultural commodity groups (or species) has resulted in commensurate changes in phylogenetic diversity.

On one hand, the aggressive expansion of a relatively small number of plant crop lineages at the expense of others in certain parts of the world–including oil crops and western cereals such as wheat and maize or other crops within the Poaceae or Fabaceae–suggest that fewer plant evolutionary lineages now makeup agricultural lands [7, 17]. This increasing dominance of a small number of commodity groups would lead to expectations of strong temporal declines in crop phylogenetic diversity globally. Yet on the other hand, the potential for different regions, especially in the tropics, to cultivate and produce an evolutionary diverse range of crops would suggest metrics of phylogenetic diversity has in fact increased drastically over the past 50 years, with patterns differing among regions.

Hypothesized changes in crop taxonomic- and phylogenetic diversity within regions (i.e. crop *α*-diversity), also leads to expectations of shifts in crop diversity among regions (i.e. crop *β*-diversity). Specifically, if detected, systematic increases in crop *α*-diversity are likely to correspond to declines in crop *β*-diversity among regions, since increasing crop diversity on farms within one region will necessarily increase the likelihood of observing similar crops on farms within other regions. This is particularly the case when new crops are being introduced from other regions, and not being derived through *in situ* crop diversification. Such patterns would indicate increasing homogenization of croplands worldwide, which would have major implications for agricultural sustainability worldwide. In particular, under conditions of declining crop *β*-diversity globally, theory and observation would predict increasing crop susceptibility to pest and pathogen outbreaks, or climatic change, at global scales [7].

Here, we employ crop production data from the Food and Agricultural Organization (FAO) [18] from 1961–2014, available for 161 plant-based commodity groups (Table A in S1 File), across 22 FAO-recognized subcontinental regions, in order to address the following questions: 1) Do agricultural regions show differing patterns of crop taxonomic and phylogenetic diversification through time? 2) Are changes in crop taxonomic and phylogenetic diversity predicted by agricultural expansion alone? 3) Do changes in crop taxonomic diversity result in commensurate change in the phylogenetic diversity of crops through time? And finally 4) how do changes in crop diversity within regions (i.e. crop *α*-diversity) impact the differences in crop diversity among regions (i.e. crop *β*-diversity)?

## Materials and methods

### Crop species identification and abundance estimates

Our analysis was based on crop production data from the Food and Agricultural Organization (FAO) from 1961–2014, which is reported for 161 plant-based commodity groups (Table A in S1 File) [18]. We extracted data on the area harvested (in ha) across 22 FAO-recognized subcontinental regions, as well as an overall global estimate. Complete data on area harvested (*n* = 54 years) was available for all 161 FAO commodity groups in all regions except for Central Asia (where data dates from 1992–2014, *n* = 23 years; S2 File).

Our analysis employed diversity metrics that relied on species-level taxonomy, by following a two-step process to identify the crop species associated with each commodity group. (Although genetic diversity is a critical aspect of agricultural diversity, it is not possible to account for cultivar or landrace level diversity empirically since the FAO does not report data beyond the species level.) First, we used FAO commodity group codes, in conjunction with the FAO Commodity List tool (www.fao.org/economic/ess/ess-standards/commodity), to identify the crop species the FAO associates with each group (Table A in S1 File). Second, the final list of crops for each commodity group was cross-referenced with the Taxonomic Name Resolution Service v. 4.0 [19], to correct inconsistencies and remove synonyms. Through this process we removed four FAO commodity groups that were not associated with any specific crop species, or associated with non-plant species. Our analysis thus relied on 157 commodity groups associated with 337 unique crop species across 233 genera and 77 families (Table A in S1 File).

Since FAO does not differentiate harvested area associated among species within commodity groups, diversity in each region-by-time combination was calculated assuming only one species per commodity group. While this approach may underestimate diversity in some instances, the only viable alternative–assuming all species within a group are present within a region–would present a stronger bias (e.g. the “Vegetable, Fresh nes” group contains 27 species; Table A in S1 File). We used area harvested as our estimate of species abundance.

### Measuring taxonomic crop alpha (*α*-) diversity

For each region-by-year combination we calculated the diversity of crops within a region (i.e. crop alpha (*α*-)) as both i) species richness (SR), or simply the number of crops present within a given region within a given year, and ii) Simpson’s diversity index (*S*_*D*_). The latter (*S*_*D*_) was calculated using the ‘vegan’ R package [20], and in short, is a metric in community ecology that accounts for both SR and the abundance of species when quantifying *α*-diversity. This index ranges from 0–1, such that an *S*_*D*_ value of 1 represents “infinite diversity” and an *S*_*D*_ value of 0 represents no diversity. For our analyses here changes in *S*_*D*_ through time can be interpreted as follows: i) increases in *S*_*D*_ within a given region through time, are interpreted as reflecting regions where overall crop *α*-diversity is comprised of a greater number of crops which are becoming more equitable in their abundances, while i) declines in *S*_*D*_ within a given region through time, are interpreted as reflecting regions where overall crop *α*-diversity is becoming dominated by a fewer number of highly abundant crop species.

### Measuring phylogenetic/ evolutionary crop alpha (*α*-) diversity

We then calculated metrics of phylogenetic/ evolutionary crop diversity that were essentially analogous to SR and *S*_*D*_, but instead take into account evolutionary diversity among crop species. This was done by first constructing a quantitative representation of the evolutionary relationships among crops (i.e. a phylogenetic tree). Our crop phylogenetic tree was based on the evolutionary relationships among all plant species, as proposed in the Angiosperm Phylogeny Group III “megatree” [21]. We then used Phylomatic [22] in order to “prune” this megatree such that our phylogenetic tree included only crops species (see Table A in S1 File). Then, we quantitatively measured the evolutionary distances among all crops (measured in millions of years of evolution) by using the BLADJ algorithm in Phylocom [23], which ascribes evolutionary ages to each different crop based on fossil records [24] as updated by [25]. Unresolved evolutionary relationships were treated as polytomies.

For each region-by-year combination we then quantified Faith’s phylogenetic diversity (PD), which is a phylogenetic analogy to SR. However, instead of representing simply the number of crop species within a given region-by-year combination, PD represents the sum of all phylogenetic branch lengths within a region-by-year sample [26]; in other words, PD represents the total millions of years of plant evolution captured by the crops growing within a given region within a given year.

We also calculated a phylogenetic analogue of *S*_*D*_, which is phylogenetic Rao’s quadratic entropy (QE_phy_) [27, 28]. A value of QE_phy_ was calculated for each region-by-year combination as:

$${QE}_{phy}=\sum_{i}\sum_{j\neq 1}{(d}_{ij}\times p_{i}p_{j})$$ (1)

where *d*_ij_ represents the phylogenetic distance between species *i* and *j*, and *p* represents the proportional abundance of species *i* and *j* within any given region-by-year grouping. For our analysis, QE_phy_ is a metric of crop *α-*diversity that is similar to *S*_*D*_, but takes into account the evolutionary relatedness among crop species. In our analysis then, i) increases in QE_phy_ through time represent regions where crop *α*-diversity is becoming defined by a larger number of crop evolutionary lineages that are more equitable in their abundances, while ii) decreases in QE_phy_ through time represent regions where a smaller number of crop evolutionary lineages are becoming more dominant in their abundances.

All crop evolutionary/ phylogenetic diversity metrics were calculated using the ‘picante’ R package [29]. In calculating both PD and QE_phy_, we had to account for the discrepancy between FAO commodity groups containing multiple species, while presenting only a single corresponding estimate of area harvested. Therefore, for each individual measurement both PD and QE_phy_ were calculated assuming only one randomly selected species per commodity group (see Table A in S1 File). This randomization was repeated 100 times, and final values of PD and QE_phy_ used in analysis for each region-by-time data point were taken as the median PD and QE_phy_ values from the randomized distributions.

### Changes in crop alpha (*α*-) diversity through time

Our preliminary analysis entailed fitting and comparing a number of linear and non-linear models, in order to evaluate patterns of crop taxonomic- and phylogenetic diversity through time (as well as total agricultural area harvested) in each region individually. For these analyses, we assumed that any metric of diversity (*D* in Eqs 2–7 below) might change following six different linear and non-linear patterns. First, we fit a linear regression model of the form:

D=a+(b×year) (2)

where *a* is the intercept and *b* represents the rate of change in *D* through time. Based on this linear model, we then fit a piecewise linear regression model using the “segmented” R package [30]. This entailed using the linear model from Eq 2, to estimate breakpoints in the relationship between *D* and year. Piecewise models were of the form:

$$D=a+b(year)+(c(year-\psi_{1})\times I(year>\psi_{1}))+(d(year-\psi_{2})\times I(year>\psi_{2}))$$ (3)

where *a* is as in Eq 2, and *b* represents the slope of the *D*-year relationship prior to the first breakpoint (ψ1). In these models, *c* represents the difference in the slope of the *D*-year relationship between the first and second segments, which therefore applies only when the first conditional indicator function (denoted by “*I*”) is true. Similarly, *d* represents the difference in slopes for the *D*-year relationship between the first, second, and third segments, which only applies when the second conditional indicator function is true. In sum, the slope of the relationship between *D* and year is equal to *b* prior to the ψ1, is equal to *b* + *c* between the ψ1and ψ2, and is equal to *b* + *c* + *d* after the ψ2. Since there may be multiple solutions to the piecewise model fitting process, all model parameters as well as overall model Akaike’s Information Criterion (AIC) and *r*^2^ value, were calculated as median values of a distribution for these parameters generated through bootstrapping with replacement (where *n* = 500 replicates) [31].

We then fit a linear model including a second-order polynomial term of the form:

$$D=a+(b\times \mathrm{year})+(c\times {\mathrm{year}}^{2})$$ (4)

where *a* and *b* are as in Eq 2, and *c* represents to the coefficient for the year^2^ term which controls the form of the parabolic curve.

We then used non-linear least square to fit a number of non-linear models, the first of which was a unimodal model of the form:

$$D=a\times b^{{\left(\mathrm{year}-c\right)}^{2}}$$ (5)

where *a*, *b*, and *c* are parameters representing the mean value of *D*, the shape of the unimodality, and the year of the peak, respectively. The next model was an asymptotic model of the form:

D=a+b×exp(‑exp(c)×year) (6)

where *a* represents the asymptote, *b* represents the difference between the *y*-intercept and the asymptote, and *c* represents the log of the rate constant. Next we fit a four-parameter logistic model of the form:

$$D=\frac{a+\left(b-a\right)}{\left(1+\exp\left(\frac{c-\mathrm{year}}{d}\right)\right)}$$ (7)

where *a* represents the minimum value of *D* (a lower asymptote), *b* represents the maximum value of *D* (the upper asymptote), and *c* and *d* represent the slope and the inflection point of the curve, respectively. Values for all parameters in Eqs 5–7 were estimated using maximum likelihood.

### Model comparisons

For all regions, we then identified the best-fit model for each *D*-by-year relationship (as well as agricultural area-by-year) using AIC, with the lowest AIC score indicating the most parsimonious model fit. Among all models (Eqs 2–7), overwhelmingly two-break piecewise models (Eqs 2 and 3) best described changes in *D* through time (Table B and Fig B in S1 File). Of the 88 regional trends in four different diversity metrics evaluated, AIC-selection indicated that piecewise models represented the most parsimonious model in 78 instances; in the 10 total cases where segmented models were not AIC-selected, differences in AIC between these models and the most parsimonious fit were low and piecewise models generally showed the second-lowest AIC value (Table B and Fig B in S1 File). Similarly, piecewise models also best explained changes in agricultural area through time in 16 instances (Table B and Fig B in S1 File). Therefore we used piecewise model fits for all *D*-year relationships, in order to facilitate further analyses.

### Regional characteristics of change in crop alpha (*α*-) diversity through time

We used parameters from piecewise models to assess and compare changes in crop *D* through time across regions as follows. First, ψ1 in Eq 3 was used to quantify the timing of the first major breakpoint in crop *D* change. Secondly, we calculated the duration of the period of major change in crop *D* through time as ψ1-ψ2 from Eq 3. Third, we calculated the change in the slope of the relationship between *D* and year that occurs at ψ1, as an indicator of both the direction and rate of change in *D* through time following the first major breakpoint (calculated as *b* + *c* from Eq 3).

We then focused more explicitly on the first breakpoints in *D*, in order to test if the timing of crop *D* change mirror patterns of agricultural expansion. This was done using linear regression analyses where the timing of the first breakpoint in agricultural area was treated as the independent variable, and the timing of the first breakpoint in *D* as the dependent variable. Similarly, we assessed if the timing of major changes in crop taxonomic- or phylogenetic diversity differed from one another. This analysis was performed using linear regression analysis, where ψ1 (Eq 3) for one measure of *D* was predicted as a function of ψ1 for any other measure of *D*. We tested if these linear models differed from a 1:1 relationship using a linear hypothesis test using the ‘linear.hypothesis’ function in the ‘car’ R package [32].

### Changes in regional differences in crop beta (*β*-) diversity through time

We used non-metric multidimensional scaling (NMDS) to assess changes in crop *β*-diversity among regions and through time. This entailed constructing a global community matrix of all region-by-year crop “communities”, and then calculating pairwise Bray-Curtis (BC) dissimilarity values among all communities as:

$${BC}_{jk}=\frac{\sum i|x_{ij}-x_{ik}|}{\sum i(x_{ij}+x_{ik})}$$ (8)

where *BC*_jk_ represents the dissimilarity between the *j*th and *k*th sample, *x*_ij_ represents the abundance (i.e., area harvested) of taxon *i* in sample *j*, and *x*_ik_ represents the abundance of taxon *i* in sample *k*. We used an Adonis test–essentially a multivariate analogue to an analysis of variance (ANOVA)–to evaluate if pairwise distances differed significantly as a function of region, year, and a region-by-year interaction. An Adonis test also returns an *r*^2^ value. Therefore in our analysis, the Adonis tests informs our statistical evaluation of i) the presence/ absence of statistical differences in crop diversity among regions, among years, and among all region-by-year combinations, as well as ii) the degree to which region or year explain differences in crop *β*-diversity among all region-by-year groups. All statistical analyses were conducted using R statistical software v 3.3.2 [33].

## Results

### Changes in crop diversity within regions

**Fig 1:**
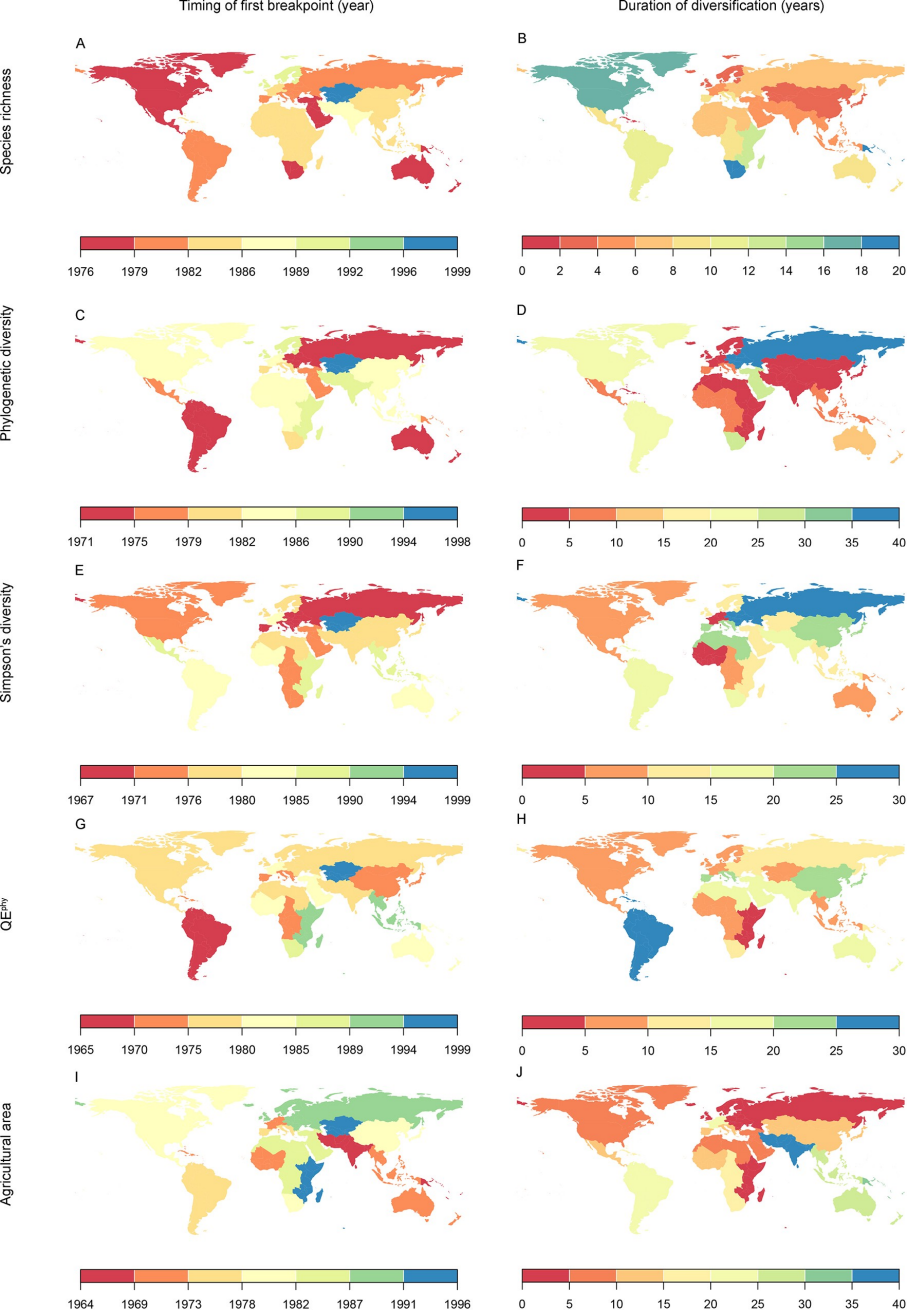
Timing of the onset and duration of changes in regional crop diversity and agricultural area across 22 FAO-defined agricultural regions. Detailed values and confidence limits surrounding them are presented in Tables C and D in S1 File.

**Fig 2:**
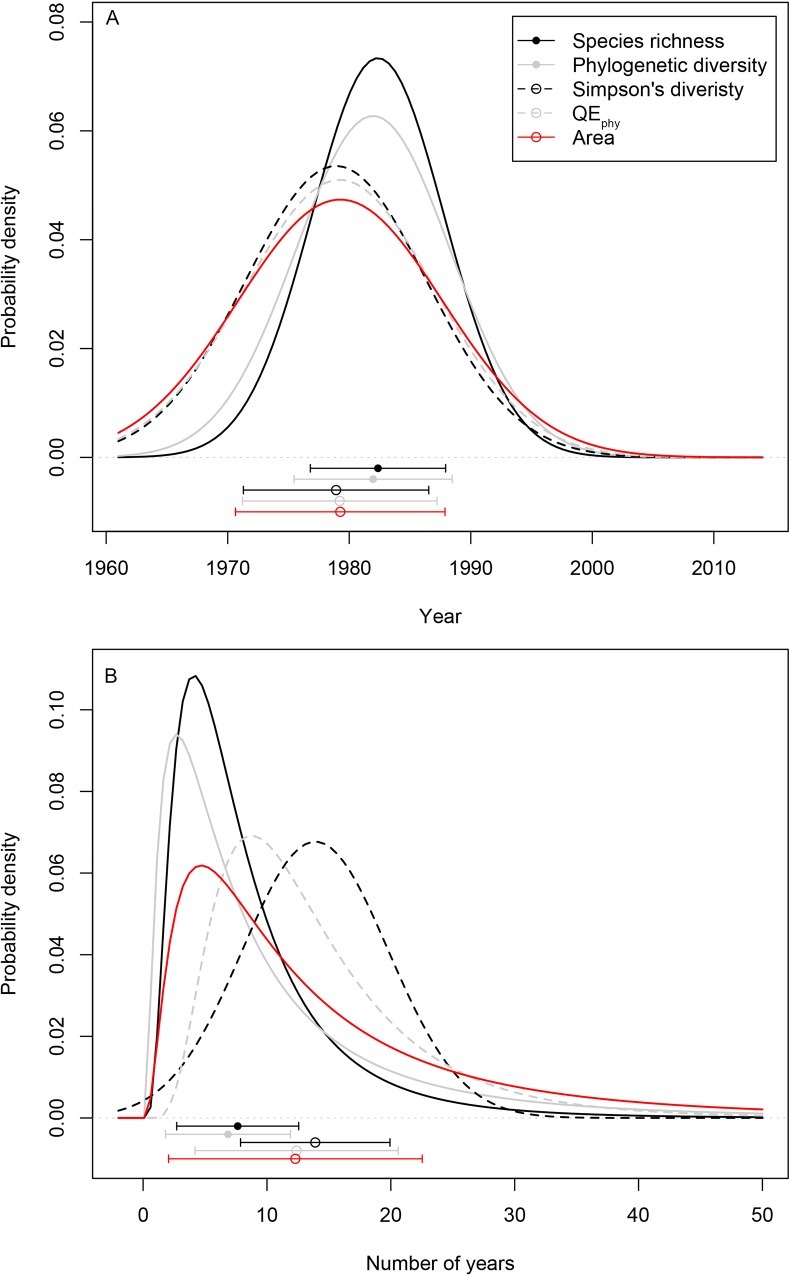
Probability distributions of two indicators of change in crop diversity and agricultural area across 22 FAO-defined agricultural regions. Panel A represents data on the timing of major change in crop diversity and agricultural area, while panel B represents data on the duration of major change in four different crop diversity metrics and agricultural area (where *n* = 22 in all cases). Region-specific values for each of these indicators, as well as a third indicator associated with the rate of change in crop diversity metrics and agricultural area, are presented in Table C in S1 File. Points below the histograms in panels A and B correspond to means ± 1 standard deviation, and medians ± 1 median absolute deviation, respectively.

Major shifts in regional crop taxonomic diversity (measured as crop species richness (SR)) and phylogenetic diversity (PD), occurred within all regions in very similar patterns: i) a period of little change in diversity from the early- through to the late 1960s, followed by ii) a period of rapid increase in diversity beginning in the late 1970s through mid-1980s, which endured for ~9.5–10.5 years on average, which then iii) ultimately stabilized in the early 1990s (Figs 1 and 2, Figs A and B and Tables C and D in S1 File).

Across all 22 regions, pronounced increases in both SR and PD began on average in 1982 (SR average = 1982.4±5.6 years (s.d.); PD average = 1982.0±6.5, Fig 2). When evaluated at a global scale, across all regions the onset of increases in crop diversity did not differ significantly between SR and PD (Fig 2), but there were some notable exceptions. Increases in crop PD began in North America 8.0 years prior to SR, while increases in PD in Eastern- and Southern Africa occurred 3.1 and 5.4 years before increases in crop SR began, respectively (Fig 1, Table D in S1 File). At the same time, increases in SR in South America and Eastern Europe were initiated at 8.5 and 10.7 years, respectively, prior to any commensurate increase in PD (Table D in S1 File).

Following the onset of crop diversity increases, regional SR then increased on average by 2.1±2.4 species year^-1^ with increases continuing for 8.4±5.4 years on average. Similarly, following the onset of crop diversity increases, across all regions PD increased by 93.7 million years year^-1^ on average; increases which were maintained for 10.7 years on average. The only exception to these patterns were three regions in Oceania which showed only subtle declines in SR of 0.3 species year^-1^ and PD of 18.9 million years year^-1^, beginning in 1979–80 and occurring for a period of ~10 years (Fig 2, Figs A and B and Table D in S1 File).

**Fig 3:**
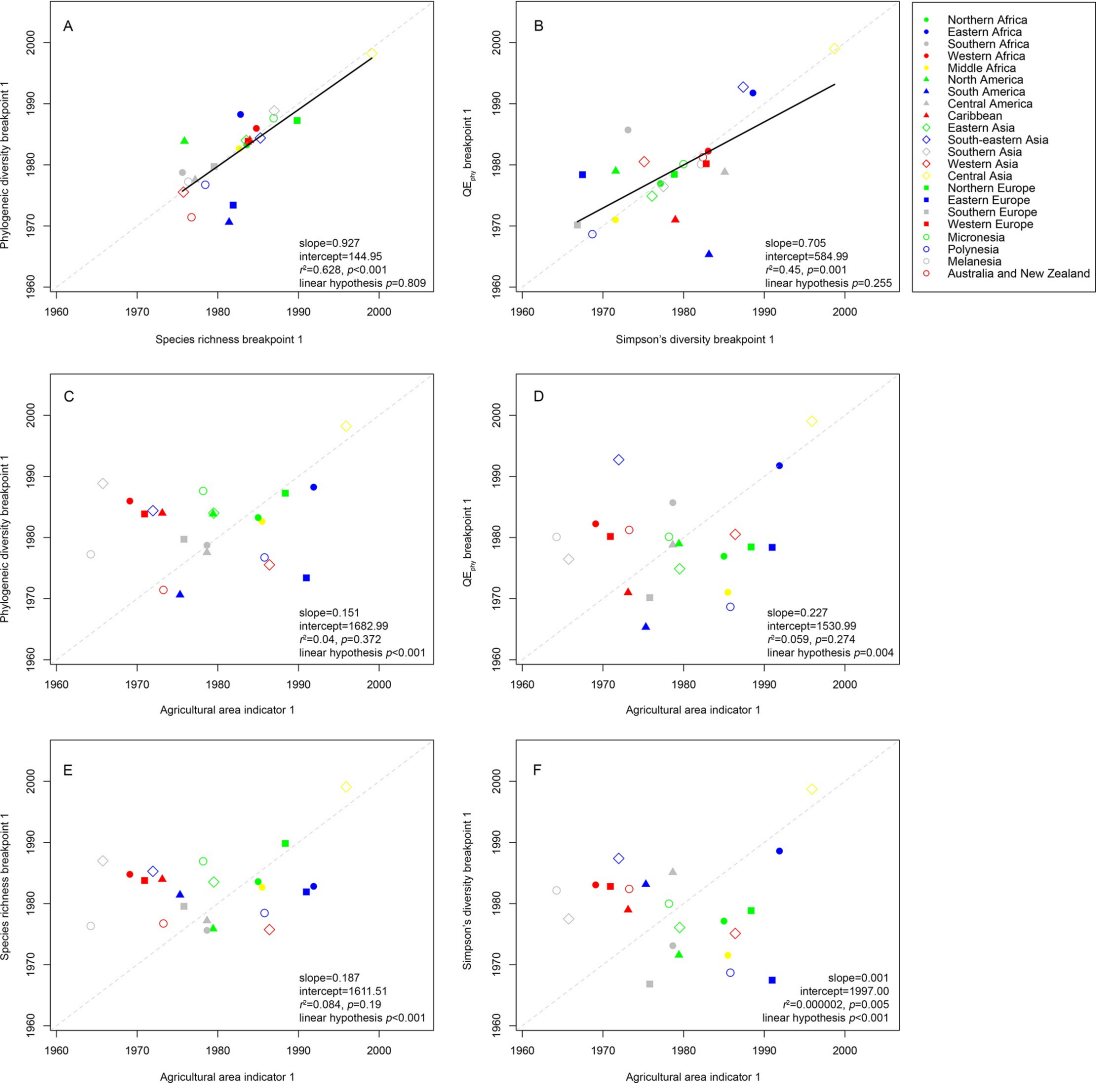
Relationships between the timing of the first breakpoint in agricultural area as compared to timing of the first breakpoint in four metrics of crop diversity across 22 FAO-defined agricultural regions. Dashed gray lines represent a 1:1 trend line, and bold black trend lines represent significant relationship among the breakpoints based on linear regression. Linear hypothesis *p*-values >0.05 indicate relationships that do not differ significantly from a 1:1 line.

When evaluated at a global scale, the timing and duration of change in crop diversity and agricultural area both increased through time (Fig 2) However, when examined more in detailed within regions, these patterns did not mirror one another. Neither the onset of increases in SR nor PD was correlated with onset of increases in agricultural expansion. The timing of major increases in agricultural area occurred anywhere from ~21 prior to, to ~11 years following, increases in SR. Similarly, the onset of sharp increases in agricultural area occurred anywhere from ~23 years prior to, to ~17 years following, increases in PD (Fig 3). Periods of increases in agricultural area tended to last longer on average (14.6±10.0), as compared to the duration of SR and PD increases (Fig 2).

Across all regions in the global dataset, six new crop commodity groups were reported between 1961 and 2014, including cassava leaves (first reported by the FAO in 1990), dry cow peas (1969), jojoba seed (1981), kiwi (1969), tallow tree seed (1984), and triticale (1975). Within regions, the largest net increases in crop SR between 1961 and 2014 were observed in Central America, where additional 30 crop commodity groups were reported from a wide range of crop types, including cereals (e.g. rye, millet, and non-specified cereals), legumes (e.g. string beans and non-specified pulses), tree and nut crops (e.g. almond, areca nut, cashew, rubber, sunflower, tea), spices (e.g. ginger, pepper, as well as nutmeg, mace, and cardamom), vegetables (e.g. artichoke, leeks and other alliaceous vegetables, spinach, taro) and, to a lesser extent, fruits (e.g., blueberry and persimmons). Alternatively, in the same time period Polynesia reported increases of only one additional crop commodity group (i.e. lettuce and chicory) and Micronesia reported only three additional crops (i.e. cucumbers and gherkins, pineapples, and plantains).

### Changes in crop composition within regions

When evaluated at a global scale, the timing of major changes in crop composition, as measured by Simpson’s diversity index (*S*_*D*_) and a phylogenetic analogue of *S*_*D*_ (Rao’s quadratic entropy; QE_phy_), was broadly similar to the timing of change in crop SR and PD (Fig 2). Across all regions on average, crop *S*_*D*_ and QE_phy_ began shifting in ~1979–1982 (Figs 1 and 2). However, when evaluated within regions, unlike SR, PD, or agricultural area, these were not necessarily periods of rapid increases. In 10 of 22 regions evaluated, this time marked a period of declines of *S*_*D*_−i.e. a period of increasing dominance of a smaller number of crop species–through much of the 1980s, over a period of 11.4±4.5 years (Figs C and D and Table D in S1 File). Similarly, nine regions showed a period of declining QE_phy_ throughout the 1980s –i.e. a period therefore defined by increasing dominance of a few evolutionary lineages–over 9.0±3.7 years on average.

In regions where the 1980s were marked by declines in *S*_*D*_ and QE_phy_ (i.e. increasing dominance of a few crop species or evolutionary lineages), this period was then followed by increases in these diversity metrics throughout the 1990s to mid-2010s (Figs C and D and Table D in S1 File). The timings of the first breakpoints in *S*_*D*_ and QE_phy_ were correlated across all regions, but the timing of the onset of increases in *S*_*D*_ or QE_phy_ was not predicted by the timing of increases in agricultural area (Fig 3).

### Changes in crop diversity among regions

**Fig 4:**
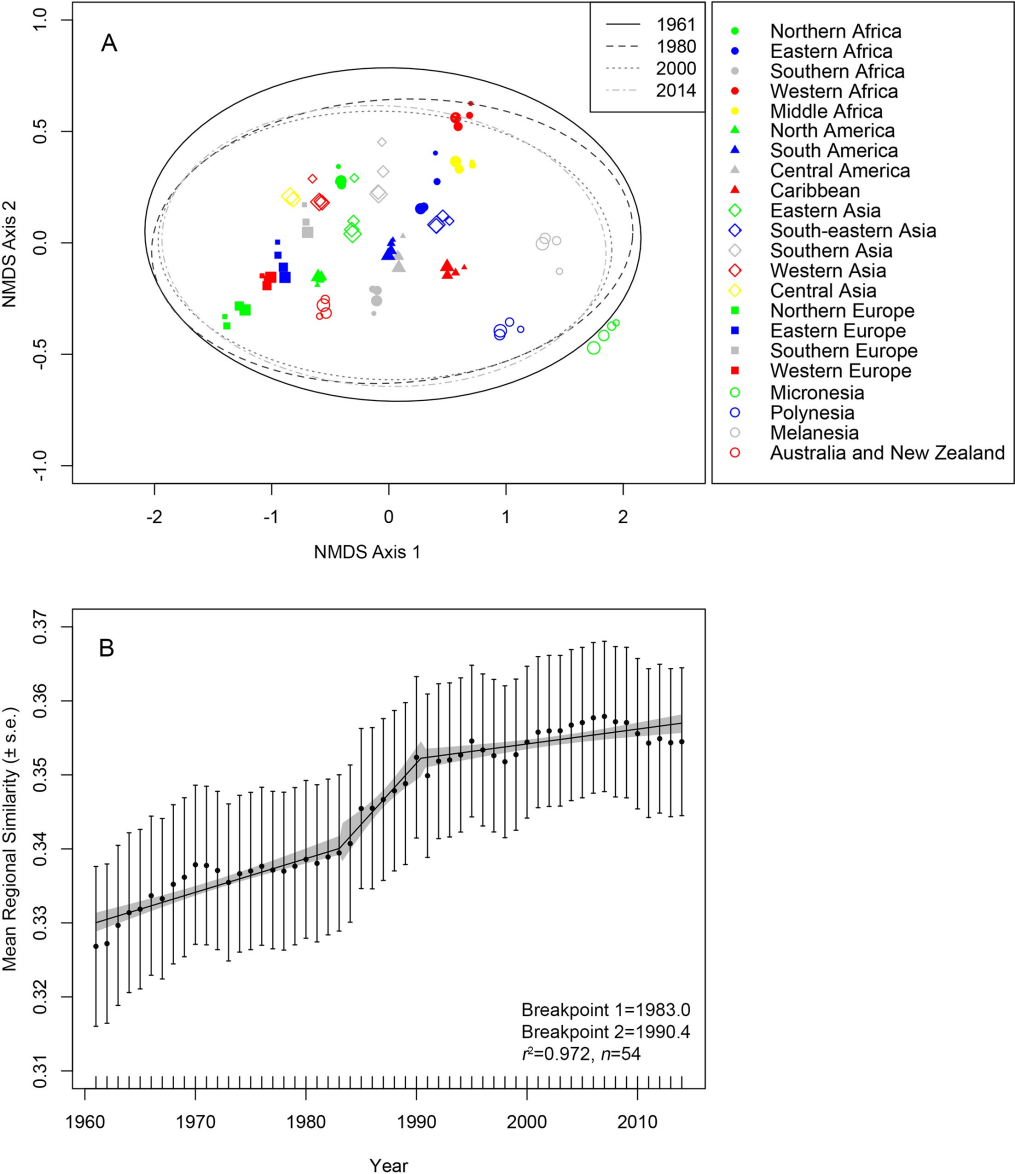
Changes in the differences in crop diversity among 22 FAO-defined agricultural regions from 1961–2014. Panel A presents a visualization of regional differences in crop *β*-diversity (calculated following a common metric in community ecology: non-metric multidimensional scaling (NMDS)). The values associated with this technique (NMDS axis 1 and NMDS axis 2) are plot and interpreted here visually as a “map of similarity”: points closer to one another are region-by-year combinations with more similar crop composition, while points further from one another are more dissimilar in crop composition. For clarity only data points from 1961, 1980, 2000, and 2014 are shown; corresponding confidence ellipses represent 1 standard deviation for each of these four years. Sizes of symbols within a region corresponding to year with 1961 represented by the smallest symbols, 1980 medium size symbols, 2000 large symbols, and 2014 the largest symbols. Panel B represents changes in regional crop similarity from 1961 to 2014. Data points represent similarity between any two regions, and trend line represents a segmented linear regression model fit to the entire dataset fit. Shaded gray bands are 95% confidence limits surrounding the model.

We detected statistically significant differences in crop diversity among regions (Adonis test *r*^2^ = 0.949, *p*<0.001; Fig 4, Fig D in S1 File). Yet when controlling for regional differences, crop *β*-diversity also changed significantly through time (Adonis test *r*^2^ = 0.01, *p*<0.001): there was a significant, though small in magnitude, trend of increasing the similarity of crops grown among different regions occurring between 1961 through to 2014 (*r*^2^ = 0.003, *p*<0.001, *n* = 11823). These subtle increases in the similarly of crops grown among regions, or alternatively significant declines in the crop diversity among regions, occurred over a ~9-year period from 1983 to 1992 (Fig 4).

## Discussion

### Trends in crop diversity within and among regions

Our analyses show that over time the taxonomic- (SR) and phylogenetic (PD) diversity of crops produced within regions has increased significantly over the past 50 years, in nearly all regions of the world (Fig A and B in S1 File). While crop diversity within regions has generally systematically increased, our results indicate that these changes lead to a weak but statistically significant signal of homogenization of agricultural lands among regions throughout the same period (Fig 4). These patterns are similar to, albeit weaker than, those observed in analyses of national-level food consumption patterns [7].

Our analyses also indicate that global scale assessments of diversity in agricultural lands (or other metrics such as food consumption patterns e.g. [7]) may miss important differences occurring at regional scales. For instance, an assessment of global averages (and associated errors) indicated overlap in the timing and duration of change in all agricultural diversity indices and area (Fig 2). Yet these same metrics differed widely when assessed at regional scales (Fig 3), indicating that any interpretations that global patterns in agricultural diversity reflect change occurring on smaller scales should be done cautiously. Indeed, moving beyond regional assessments by employing our analytical framework at the national-level, and then linking these patterns with explicit local environmental or socio-economic data or policy, represent a next step in elucidating smaller-scale changes in crop diversity in greater detail.

Our study also shows that increases in regional crop diversity, and associated declines in regional differences in crop diversity, are not explained by patterns of agricultural expansion alone (Fig 3). This indicates that the complex interaction of factors leading to certain crops being grown across regions–such as crop-specific subsidies, international trade markets, population growth and per capita food demand, or environmental change e.g. [10, 11, 12]–are not necessarily the same that drive agricultural expansion. For example, much of the literature evaluating linkages between the political economy of food systems and crop diversity stems from Central America and the Caribbean, where research has demonstrated how international trade agreements have influenced the diversity of crops grown on farms independently of agricultural expansion e.g. [11, 34]. Within this and nearby regions, the North American Free Trade Agreement (NAFTA) and other initiatives such as the Caribbean Basin Initiative in the mid-1980s, resulted in major increases in the number of crops grown within the national agricultural portfolio of Central American countries. These changes specifically entailed introduction of palm oil, spices, fruits, and multiple “winter vegetables” into agricultural lands [34]; changes which correspond closely to the onset and duration of crop diversification in Central America observed throughout the early to mid-1980s (Fig 1).

However, such conclusions must be interpreted within the context of data limitations. Specifically, increases in agricultural diversity here, quantified as changes in the crop species associated FAO-defined commodity groups, may underestimate the number of cultivars, varieties, and landraces that comprise agricultural lands. For instance the NAFTA established in the mid-1990s, and other trade liberalization initiatives in the region, have demonstrably reduced the genetic diversity of maize across Central America on existing croplands [35]. Comparative evaluation of hypotheses that predict shifts in crop diversity as a function of specific policy interventions *vs*. other environmental and socio-economic change would be key in addressing the causes of change in crop diversity further, but is beyond the scope of our analysis here.

In nearly all regions a larger number of crops are becoming more abundant in their contributions to agricultural lands; a trend supported here by consistent increases in *S*_*D*_ and QE_phy_ over the past half century (Fig 1, Table D in S1 File). Only South America, and to a lesser extent Eastern Asia and Western Europe, showed evidence of increasing dominance of certain crops or phylogenetic lineages (Fig B and Table D in S1 File). In the unique case of South America, a trend of reduced *S*_*D*_ and QE_phy_ is reflective of massive soybean expansion from ~260,000 to 55.7 million ha between 1961–2014 (Table D in S1 File).

### Implications for agricultural diversity and sustainability

Given the scale of our analyses, increases in the diversity of crops grown within regions observed here is likely not reflective of on-farm cultivation or conservation of crop varieties and landraces, which is often most notably observed on small-scale farms [11, 36]. Nor are the trends reported here likely driven by *in situ* domestication of new commercial crop species, since much of this occurred prior to ~4000–2000 years ago [2]. Instead, patterns reported here are likely attributable to increases in large-scale movement of commercial crops, specifically those being grown in conventional intensive management systems outside of their regions of origin [3].

In that sense, our results should not be interpreted as support that actual farms worldwide are shifting towards more diverse agroecosystems *sensu* [37, 38]. The environmental or socio-economic benefits of diverse agroecosystems *vs*. intensive monoculture, such as increased crop resilience to climate change or enhanced nutrient-use efficiency, are realized at farm- or agricultural landscape scales where crops are interacting with one another or other non-crop species [37–40]. Similarly, the benefits of increased crop diversity in terms of greater nutritional quality or increased food sovereignty are also largely realized at sub-regional or household scales [41] but see [42]. So while here we contribute towards finer spatial-scale analyses of crop diversity, by moving from global assessments of crop commodity group diversity to crop species- and phylogenetic diversity assessments on regional scales, considerable work remains in order moving towards even finer scale analyses of changes in crop diversity.

In its current form, our analyses can play an important role in setting and measuring global priorities for agricultural sustainability and diversity. Specifically SDG 2 Target 2.5 calls for the conservation of a “…genetic diversity of seeds, (and) cultivated plants…”: initiatives which are progressively focusing on both taxonomic and phylogenetic diversity of crops and their wild relatives, as well as intraspecific diversity within major crop lineages [43, 44]. The crop taxonomic- and phylogenetic diversity values presented here could be used as a baseline for such crop conservation initiatives, since questions surrounding “how much diversity is enough” are central to such sustainability initiatives. Similarly, SDG 2 Target 2.4 calls for “…resilient agricultural practises…” that confer a multitude of ecosystem services beyond yield alone. Diversified agroecosystems that incorporate multiple crop species are key in meeting this target [45], however, political support for such systems remain limited [46, 47]. Our analyses suggests that at regional scales, diversified polyculture assemblages would also be critical in addressing both the trend towards, and consequences of, increasing homogenization in agricultural systems globally.

### Conclusions

The Anthropocene epoch is a time marked by major shifts in plant and animal biogeography, mediated by both deliberate and accidental human-caused species movement. Our data provides evidence that the 1970s-80s marked a widespread period of major increases in the taxonomic- and phylogenetic diversity of crops grown within nearly all regions of the world. And while regional differences in crop species pools persist, there is evidence of a trend towards greater homogeneity in the crops being grown in agricultural lands across regions. In basing our analysis on FAO data, the trends observed here most likely reflect of how environmental and socio-economic conditions influence species-level diversity of crops being grown in large-scale, industrial agricultural systems. Evaluating temporal changes in crop diversity at the landscape-, farm-, or household levels are ultimately critical for understanding how crop diversity can be enhanced to ensure both human well-being and agricultural sustainability.

## Supporting information

S1 File Supplementary information for Martin et al. 2019. Supplementary information for this article contained in this file includes additional statistical methodology and results, five supplementary tables, and eight supplementary figures.
(DOCX)

S2 File Data file associated with Martin et al. 2019. Base data file retrieved from the Food and Agricultural Organization data repository [18] employed in the analysis by Martin et al. 2019.
(XLSX)
