# Supplementary material for: Regional and global shifts in crop diversity through the Anthropocene
Source: PLoS One. 2019 Feb 6;14(2):e0209788. doi: 10.1371/journal.pone.0209788 (PMC6364885; doi:10.1371/journal.pone.0209788)
Supplement: S1 File — Supplementary information for this article contained in this file includes additional statistical methodology and results, five supplementary tables, and eight supplementary figures. (DOCX) [file pone.0209788.s001.docx]

**Supplementary Information for:** Regional and global shifts in crop diversity through the Anthropocene.

**Supplementary methods**

**Phylogenetic structure of regional crop species pools**

To evaluate phylogenetic community structure of regional crop species pools, we calculated mean pairwise distances (MPD) among crops within regions [1]. All MPD values were calculated as abundance-weighted indices, using agricultural area as the index of abundance. For MPD analyses, we employed a null model approach to test for significant change in phylogenetic community structure through time, as compared to a random expectation [2]. Null models of the phylogenetic structure for each region-by-year community, were generated by assigning each commodity group a randomly assigned species from the entire global species pool of crops 999 times, and recalculating the MPD. We then calculated standardized effects sizes (SES) as:

$\text{SES}=(\chi_{obs}-\chi_{null})/\text{SD}_{null}$ (1)

where *χ*_obs_ represents the observed MPD, *χ*_null_ represents a mean randomized MPD value, and *SD*_null_ represents the standard deviation of the simulated MPD values. Positive SES values for any region at a particular year indicates crop species pools that are phylogenetically over-dispersed (compared to a random expectation), while negative SES values indicate crop species pools that are phylogenetically clustered (as compared to a random expectation); regional crop species pools were considered significantly over-dispersed or clustered when χ_obs_ fell within the lower- or upper 2.5% of χ_null_ values, respectively [2]. In these analyses we also had to account for FAO groups containing multiple species. Therefore, all SES values reported and interpreted here are the mean SES values derived by randomly assigning a single species (and in turn, phylogenetic position) to each commodity group, and then performing the complete null model analysis 100 times.

To assess changes in SES as a function of time and region we used an analysis of covariance (ANCOVA), including a region-by-time interaction term. For these analyses, we were largely seeking to evaluate how regions differed from one another, and with respect to global trends. Therefore, global trends in SES were also calculated using the FAO global dataset, which were in turn set as the reference category in the ANCOVA model.

**Supplementary results**

**Crop phylogenetic structure**

Analysis of global data indicates an overall significant increase in MPD through time. Also at a global scale, there is a significant temporal trend towards more phylogenetically diverse and less clustered regional crop profiles (S1 File Table E). No regions at any year showed significant phylogenetic over-dispersion (S1 File Fig A and Table E). Phylogenetic clustering decreased through time in 14 regions however, the other eight regions (which were smaller in terms of agricultural area) did show evidence of temporal trend toward more phylogenetically clustered crops than expected by chance alone (S1 File Fig A and Table E). Notably, two regions in close proximity to one another showed the most drastic differences in trends of phylogenetic change through time: Central America and the Caribbean showed two of the strongest trends towards greater phylogenetic clustering through time, while South and North America showed two of the strongest trends towards less phylogenetic clustering (S1 File Fig A and Table E).

**Table A. Commodity groups recognized by the Food and Agricultural Organization of the United Nations and associated crop species taxonomy.** The process by which species within commodity groups were identified is specified in detail in the main text (see Methods in main text), and the notation “nes” for a commodity group denotes “Not elsewhere specified.”

| **FAO commodity group** | **FAO group code** | **Family** | **Species** | **Authority** | **Notes** |
| --- | --- | --- | --- | --- | --- |
| Agave fibres nes | 800 | Asparagaceae | *Agave americana* | L. |  |
| Agave fibres nes | 800 | Asparagaceae | *Agave angustifolia* | Haw. | FAO lists this as *Agave letonae* F.W.Taylor ex Trel. Which is a snynonym for a variety of *A*. *angustifolia* |
| Agave fibres nes | 800 | Asparagaceae | *Agave cantala* | Roxb. |  |
| Agave fibres nes | 800 | Asparagaceae | *Agave fourcroydes* | Lemaire |  |
| Agave fibres nes | 800 | Asparagaceae | *Agave lecheguilla* | Torr. |  |
| Agave fibres nes | 800 | Asparagaceae | *Furcraea foetida* | (L.) Haw. | FAO lists this as synonym *Agave foetida* |
| Almonds, with shell | 221 | Rosaceae | *Prunus dulcis* | (Mill.) D.A. Webb | FAO lists *P*. *amygdalus*, *P*. *communis*, and *Amygdalus communis*, but these are all synonyms for *P*. *dulcis* (as per USDA) |
| Anise, badian, fennel, coriander | 711 | Apiaceae | *Carum carvi* | L. |  |
| Anise, badian, fennel, coriander | 711 | Apiaceae | *Coriandrum sativum* | L. |  |
| Anise, badian, fennel, coriander | 711 | Apiaceae | *Cuminum cyminum* | L. |  |
| Anise, badian, fennel, coriander | 711 | Apiaceae | *Foeniculum vulgare* | Mill. |  |
| Anise, badian, fennel, coriander | 711 | Schisandraceae | *Illicium verum* | Hook. f. |  |
| Anise, badian, fennel, coriander | 711 | Cupressaceae | *Juniperus communis* | L. |  |
| Anise, badian, fennel, coriander | 711 | Apiaceae | *Pimpinella anisum* | L. |  |
| Apples | 515 | Rosaceae | *Malus pumila* | Mill. | FAO also lists *Malus communis*, which is a synonym for this species |
| Apples | 515 | Rosaceae | *Malus sylvestris* | (L.) Mill. | FAO also lists *Pyrus malus*, which is a synonym for this species |
| Apricots | 526 | Rosaceae | *Prunus armeniaca* | L. |  |
| Areca nuts | 226 | Arecaceae | *Areca catechu* | L. |  |
| Artichokes | 366 | Asteraceae | *Cynara cardunculus var. scolymus* | L. |  |
| Asparagus | 367 | Asparagaceae | *Asparagus officinalis* | L. |  |
| Avocados | 572 | Lauraceae | *Persea americana* | Mill. |  |
| Bambara beans | 203 | Fabaceae | *Vigna subterranea* | (L.) Verdc. |  |
| Bananas | 486 | Musaceae | *Musa acuminata* | Colla | FAO lists *M*. *sapientum*, which is a synonym for a hybrid between *M*. *acuminata* and *M*. *balbisiana*; FAO also lists *M*. *cavendishii* which is a synonym for *M*. *acuminata* |
| Bananas | 486 | Musaceae | *Musa balbisiana* | Colla |  |
| Bananas | 486 | Musaceae | *Musa nana* | Parham, non Lour. |  |
| Barley | 44 | Poaceae | *Hordeum disticum* | L. |  |
| Barley | 44 | Poaceae | *Hordeum hexasticum* | L. |  |
| Barley | 44 | Poaceae | *Hordeum vulgare* | L. |  |
| Bastfibres, other | 782 | Malvaceae | *Abroma augusta* | (L.) L. f. |  |
| Bastfibres, other | 782 | Malvaceae | *Abutilon avicennae* | Medik. |  |
| Bastfibres, other | 782 | Fabaceae | *Crotalaria juncea* | L. |  |
| Bastfibres, other | 782 | Malvaceae | *Hibiscus cannabinus* | L. |  |
| Bastfibres, other | 782 | Malvaceae | *Hibiscus sabdariffa* | L. |  |
| Bastfibres, other | 782 | Malvaceae | *Urena lobata* | L. |  |
| Bastfibres, other | 782 | Malvaceae | *Urena sinuata* | L. |  |
| Beans, dry | 176 | Fabaceae | *Phaseolus coccineus* | L. |  |
| Beans, dry | 176 | Fabaceae | *Phaseolus lunatus* | L. |  |
| Beans, dry | 176 | Fabaceae | *Phaseolus vulgaris* | L. |  |
| Beans, dry | 176 | Fabaceae | *Vigna aconitifolia* | (Jacq.) Marechal |  |
| Beans, dry | 176 | Fabaceae | *Vigna aconitifolia* | (Jacq.) Marechal | Previously *Phaselous aconitifolius* or *P*. *aconitifolia* (which are both listed by FAO, but have been removed here) |
| Beans, dry | 176 | Fabaceae | *Vigna angularis* | (Willd.) Ohwi & Ohashi |  |
| Beans, dry | 176 | Fabaceae | *Vigna mungo* | (L.) Hepper | Previously *Phaselous mungo* (which is listed by FAO, but has been removed here) |
| Beans, dry | 176 | Fabaceae | *Vigna radiata* | (L.) R. Wilczek | Same as *Phaselous aureus* (which is listed by FAO, but has been removed here) |
| Beans, dry | 176 | Fabaceae | *Vigna umbellata* | (Thunb.) Ohwi & H. Ohashi | Previously *Phaselous calcaratus* (which is listed by FAO, but has been removed here) |
| Beans, green | 414 | Fabaceae | *Phaseolus coccineus* | L. | FAO only lists *Phaseolus* spp. and *Vigna* spp. in this commodity group; however, since it appears to be very similar to the commodity group 176, the species from that group are ascribed to this group (414) as well |
| Beans, green | 414 | Fabaceae | *Phaseolus lunatus* | L. |  |
| Beans, green | 414 | Fabaceae | *Phaseolus vulgaris* | L. |  |
| Beans, green | 414 | Fabaceae | *Vigna aconitifolia* | (Jacq.) Marechal |  |
| Beans, green | 414 | Fabaceae | *Vigna aconitifolia* | (Jacq.) Marechal |  |
| Beans, green | 414 | Fabaceae | *Vigna angularis* | (Willd.) Ohwi & Ohashi |  |
| Beans, green | 414 | Fabaceae | *Vigna mungo* | (L.) Hepper |  |
| Beans, green | 414 | Fabaceae | *Vigna radiata* | (L.) R. Wilczek |  |
| Beans, green | 414 | Fabaceae | *Vigna umbellata* | (Thunb.) Ohwi & H. Ohashi |  |
| Berries nes | 558 | Ericaceae | *Gaylussacia baccata* | (Wangenh.) K. Koch | FAO cites only *Gaylussacia* spp. for huckleberry and dangleberry; this species corresponds to the most common (black) huckleberry |
| Berries nes | 558 | Ericaceae | *Gaylussacia frondosa* | (L.) Torr. & A. Gray ex Torr. | FAO cites only *Gaylussacia* spp. for huckleberry and dangleberry; this species corresponds to dangleberry |
| Berries nes | 558 | Moraceae | *Morus alba* | L. |  |
| Berries nes | 558 | Moraceae | *Morus nigra* | L. |  |
| Berries nes | 558 | Moraceae | *Morus rubra* | L. |  |
| Berries nes | 558 | Myrtaceae | *Myrtus communis* | L. |  |
| Blueberries | 552 | Ericaceae | *Vaccinium corymbosum* | L. |  |
| Blueberries | 552 | Ericaceae | *Vaccinium myrtillus* | L. |  |
| Brazil nuts, with shell | 216 | Lecythidaceae | *Bertholletia excelsa* | Humb. & Bonpl. |  |
| Broad beans, horse beans, dry | 181 | Fabaceae | *Vicia faba* | L. | FAO also lists several varieties |
| Buckwheat | 89 | Polygonaceae | *Fagopyrum esculentum* | Moench |  |
| Cabbages and other brassicas | 358 | Brassicaceae | *Brassica oleracea* | L. | FAO notes this entails all varieties except *B*. *botrytis* |
| Cabbages and other brassicas | 358 | Brassicaceae | *Brassica rapa* | L. | FAO lists this as *B*. *chihensis* which is a synonym for a subspecies of *B*. *rapa* |
| Canary seed | 101 | Poaceae | *Phalaris canariensis* | L. |  |
| Carobs | 461 | Fabaceae | *Ceratonia siliqua* | L. |  |
| Carrots and turnips | 426 | Apiaceae | *Daucus carota subsp. sativus* | Hoffm. |  |
| Cashew nuts, with shell | 217 | Anacardiaceae | *Anacardium occidentale* | L. |  |
| Cashewapple | 591 | Anacardiaceae | *Anacardium occidentale* | L. |  |
| Cassava | 125 | Euphorbiaceae | *Manihot esculenta* | Crantz | FAO also lists *M*. *dulcis* and *M*. *utilissima* as separate species, but they appear to be synonyms |
| Cassava | 125 | Euphorbiaceae | *Manihot palmata* | Müll.Arg. |  |
| Cassava leaves | 378 | Euphorbiaceae | *Manihot esculenta* | Crantz | FAO also lists *M*. *utilissima* as separate species, but they appear to be synonyms |
| Castor oil seed | 265 | Euphorbiaceae | *Ricinus communis* | L. |  |
| Cauliflowers and broccoli | 393 | Brassicaceae | *Brassica oleracea* | L. | FAO lists *B*. *oleracea* var. *botrytis*, and subvarieties *cauliflora* and *cymosa* |
| Cereals, nes | 108 | Amaranthaceae | *Amaranthus caudatus* | L. |  |
| Cereals, nes | 108 | Chenopodiaceae | *Chenopodium pallidicaule* | Aellen |  |
| Cereals, nes | 108 | Poaceae | *Coix lacryma jobi* | L. |  |
| Cereals, nes | 108 | Poaceae | *Eragrostis abyssinica* | (Jacq.) Link | Synonym is *Eragrostis tef* (Zuccagni) Trotter |
| Cereals, nes | 108 | Poaceae | *Zizania aquatica* | L. |  |
| Cherries | 531 | Rosaceae | *Prunus avium* | (L.) L. | Synonym is *Cerasus avium* (listed on FAO reference set as a separate species, so not included here); also listed are varieties *juliana* and *duracina* |
| Cherries, sour | 530 | Rosaceae | *Prunus cerasus* | L. | FAO lists *Cerasus acida*, but *C*. *acida* is commonly listed as a synonym for *P*. *avium* (e.g. in FAO group number 531). Removed from this FAO group |
| Chestnut | 220 | Fagaceae | *Castanea sativa* | Mill. | FAO also lists *Castanea vesca* Gaertn. and *C*. *vulgaris* as species, but these are synonym for *C*. *sativa* so are not included here |
| Chick peas | 191 | Fabaceae | *Cicer arientinum* | L. |  |
| Chicory roots | 459 | Asteraceae | *Cichorium intybus var. sativum* | (Bisch.) Janch. | FAO lists *C*. *sativum* and *C*. *intybus* as separate species, but these species are *C*. *intybus* var. *sativum* (a variety bred explicitly for roots) |
| Chillies and peppers, dry | 689 | Solanaceae | *Capsicum annuum* | L. | FAO lists *C*. *frutescens* as a separate species, but it is a synonym for *C*. *annuum* var. *annuum* so is removed here |
| Chillies and peppers, dry | 689 | Myrtaceae | *Pimenta officinalis* | Lindl. |  |
| Chillies and peppers, green | 401 | Solanaceae | *Capsicum annuum* | L. | FAO lists *C*. *frutescens* as a separate species, but it is a synonym for *C*. *annuum* var. *annuum* so is removed here |
| Chillies and peppers, green | 401 | Myrtaceae | *Pimenta officinalis* | Lindl. |  |
| Cinnamon (canella) | 693 | Lauraceae | *Cinnamomum cassia* | Nees ex Blume | Common synonym is *C*. *aromaticum* Nees |
| Cinnamon (canella) | 693 | Lauraceae | *Cinnamomum verum* | J. Presl | FAO lists *C*. *zeylanicum*but this appears to be a less commonly used synonym for *C*. *verum* |
| Cloves | 698 | Myrtaceae | *Syzygium aromaticum* | (L.) Merr. & L.M. Perry | FAO lists *E*. *caryophyllata* and *C*. *aromaticus* as species, but these are synonyms for *S*. *aromaticum* so are not included here |
| Cocoa, beans | 661 | Malvaceae | *Theobroma cacao* | L. |  |
| Coconuts | 249 | Arecaceae | *Cocos nucifera* | L. |  |
| Coffee, green | 656 | Rubiaceae | *Coffea arabica* | L. |  |
| Coffee, green | 656 | Rubiaceae | *Coffea canephora* | Pierre ex Froehner | FAO lists *C*. *robusta* but this is a synonym for *C*. *canephora* so is removed here |
| Coffee, green | 656 | Rubiaceae | *Coffea liberica* | W. Bull ex Hiern. |  |
| Coir | 813 | Arecaceae | *Cocos nucifera* | L. |  |
| Cow peas, dry | 195 | Fabaceae | *Vigna unguiculata* | (L.) Walp. | FAO lists *V*. *sinensis*, but this is a synonym for a subspecies of *V*. *ungiculata* so is not included here. FAO also lists *Dolichos sinensis*, but this is a synonym for a subspecies of *V*. *ungiculata* so is not included here |
| Cranberries | 554 | Ericaceae | *Vaccinium macrocarpon* | Aiton |  |
| Cranberries | 554 | Ericaceae | *Vaccinium oxycoccus* | L. |  |
| Cucumbers and gherkins | 397 | Cucurbitaceae | *Cucumis sativus* | L. |  |
| Currants | 550 | Grossulariaceae | *Ribes nigrum* | L. |  |
| Currants | 550 | Grossulariaceae | *Ribes rubrum* | L. |  |
| Dates | 577 | Arecaceae | *Phoenix dactylifera* | L. |  |
| Eggplants (aubergines) | 399 | Solanaceae | *Solanum melongena* | L. |  |
| Fibre crops nes | 821 | Asparagaceae | *Furcraea gigantea* | (L.) Haw. |  |
| Fibre crops nes | 821 | Asparagaceae | *Furcraea macrophylla* | Baker |  |
| Fibre crops nes | 821 | Poaceae | *Lygeum spartum* | Loefl. ex L. |  |
| Fibre crops nes | 821 | Bromeliaceae | *Neoglaziovia variegata* | (Arruda da Camara) Mez |  |
| Fibre crops nes | 821 | Xanthorrhoeaceae | *Phormium tenax* | J.R. Forst. & G. Forst. |  |
| Fibre crops nes | 821 | Asparagaceae | *Samuela carnerosana* | auct. non Trel. | Important synonym is *Yucca faxoniana* (Trel.) Sarg. |
| Fibre crops nes | 821 | Asparagaceae | *Sansevieria spp* | Thunb. | FAO does not list species here |
| Fibre crops nes | 821 | Poaceae | *Stipa tenacissima* | L. |  |
| Figs | 569 | Moraceae | *Ficus carica* | L. |  |
| Flax fibre and tow | 773 | Linaceae | *Linum usitatissimum* | L. | FAO does not list any species for this group, so this taxonomy represents flax |
| Fonio | 94 | Poaceae | *Digitaria exilis* | (Kippist) Stapf |  |
| Fonio | 94 | Poaceae | *Digitaria iburua* | Stapf |  |
| Fruit, citrus nes | 512 | Rutaceae | *Citrus bergamia* | Risso & Poit. | Synonyms are *Citrus* x *aurantium* L. subsp. *bergamia* (Risso & Poit.) Wight & Arn. ex Engl. |
| Fruit, citrus nes | 512 | Rutaceae | *Citrus medica* | L. | FAO lists this is *Citrus medica* var. *cedrata* |
| Fruit, citrus nes | 512 | Rutaceae | *Citrus myrtifolia* | Raf. | Not listed on USDA taxonomy checker |
| Fruit, citrus nes | 512 | Rutaceae | *Fortunella japonica* | (Thunb.) Swingle |  |
| Fruit, fresh nes | 619 | Ericaceae | *Arbutus unedo* | L. |  |
| Fruit, fresh nes | 619 | Annonaceae | *Asimina triloba* | (L.) Dunal |  |
| Fruit, fresh nes | 619 | Rosaceae | *Crataegus azarolus* | L. |  |
| Fruit, fresh nes | 619 | Rosaceae | *Eriobotrya japonica* | (Thunb.) Lindl. |  |
| Fruit, fresh nes | 619 | Sapindaceae | *Litchi chinensis* | Sonn. | FAO lists *Nephelium litchi* Cambess. which is a synonym for *L*. *chihensis* |
| Fruit, fresh nes | 619 | Rosaceae | *Mespilus germanica* | L. |  |
| Fruit, fresh nes | 619 | Cactaceae | *Opuntia ficus-indica* | (L.) Mill. |  |
| Fruit, fresh nes | 619 | Lythraceae | *Punica granatum* | L. |  |
| Fruit, fresh nes | 619 | Rosaceae | *Rosa spp* | L. |  |
| Fruit, fresh nes | 619 | Caprifoliaceae | *Sambucus nigra* | L. |  |
| Fruit, fresh nes | 619 | Rosaceae | *Sorbus aucuparia* | L. |  |
| Fruit, fresh nes | 619 | Rosaceae | *Sorbus domestica* | L. |  |
| Fruit, fresh nes | 619 | Fabaceae | *Tamarindus indica* | L. |  |
| Fruit, fresh nes | 619 | Caricaceae | *Vasconcellea* x *heilbornii* | (V.M.Badillo) V.M.Badillo | FAO lists this as *Carica pentagona* |
| Fruit, fresh nes | 619 | Rhamnaceae | *Zizyphus zizyphus* | L. (Karst.) | FAO lists this as *Zizyphus jujuba* Mill. (synonym) |
| Fruit, pome nes | 542 | NA | NA | NA | FAO does not list any specific taxonomy with this group ("Other pome fruit not separately identified. In some countries apples, pears and quinces are reported under this general category.") |
| Fruit, stone nes | 541 | NA | NA | NA | FAO does not list any specific taxonomy with this group: ("Other stone fruit not separately identified. In some countries, apricots, cherries, peaches, nectarines and plums are reported under this general category.") |
| Fruit, tropical fresh nes | 603 | Annonaceae | *Annona cherimola* | Mill. | FAO lists this species simply as "*Annona* spp.", but then lists "cherimoya" as the corresponding product (which corresponds to this species as per USDA) |
| Fruit, tropical fresh nes | 603 | Annonaceae | *Annona reticulata* | L. | FAO lists this species under "*Annona* spp.", but then lists "custard apple" as the corresponding product (which corresponds to this species as per USDA) |
| Fruit, tropical fresh nes | 603 | Moraceae | *Artocarpus altilis* | (Parkinson) Fosberg | FAO lists this as *Artocarpus incise* which is a synonym for this species |
| Fruit, tropical fresh nes | 603 | Moraceae | *Artocarpus heterophyllus* | Lam. | FAO lists the synonym *Artocarpus integrifolius* auct. |
| Fruit, tropical fresh nes | 603 | Oxalidaceae | *Averrhoa carambola* | L. |  |
| Fruit, tropical fresh nes | 603 | Sapotaceae | *Calocarpum mammosum* | (L.) P. Royen |  |
| Fruit, tropical fresh nes | 603 | Sapotaceae | *Chrysophyllum cainito* | L. | FAO lists this simply as "*Chrusophyllum* spp", but this secies corresponds to the "star apple" and "cainito" commonodies that are listed |
| Fruit, tropical fresh nes | 603 | Sapindaceae | *Dimocarpus longan* | Lour. | FAO lists the synonym *Nephelium longan* (Lour.) Hook. |
| Fruit, tropical fresh nes | 603 | Malvaceae | *Durio zibethinus* | Murray |  |
| Fruit, tropical fresh nes | 603 | Myrtaceae | *Feijoa sellowiana* | (O. Berg) O. Berg |  |
| Fruit, tropical fresh nes | 603 | Clusiaceae | *Garcinia mangostana* | L. |  |
| Fruit, tropical fresh nes | 603 | Clusiaceae | *Mammea americana* | L. |  |
| Fruit, tropical fresh nes | 603 | Sapotaceae | *Manilkara zapota* | (L.) P. Royen | FAO lists the synonym *Achras zapota* L. |
| Fruit, tropical fresh nes | 603 | Sapindaceae | *Nephelium lappaceum* | L. |  |
| Fruit, tropical fresh nes | 603 | Passifloraceae | *Passiflora edulis* | Sims |  |
| Fruit, tropical fresh nes | 603 | Anacardiaceae | *Prunus umbellata* | Elliot | FAO lists this species simply under "*Spondias* spp, but then lists "hog plum" as the corresponding products |
| Fruit, tropical fresh nes | 603 | Myrtaceae | *Psidium guajava* | L. |  |
| Fruit, tropical fresh nes | 603 | Solanaceae | *Solanum quitoense* | Lam. |  |
| Fruit, tropical fresh nes | 603 | Anacardiaceae | *Spondias mombin* | L. | FAO lists this species as *Spondias* spp, but then lists "mombin" as the corresponding product. |
| Fruit, tropical fresh nes | 603 | Anacardiaceae | *Spondias purpurea* | L. | FAO lists this species simply under *Spondias* spp, but then lists "mombin" as the corresponding products. |
| Garlic | 406 | Liliaceae | *Allium sativum* | L. |  |
| Ginger | 720 | Zingiberaceae | *Zingiber officinale* | Roscoe |  |
| Gooseberries | 549 | Grossulariaceae | *Ribes uva-crispa* | L. | FAO lists the synonym *Ribes grossularia* L. |
| Grain, mixed | 103 | NA | NA | NA | FAO indicates this includes "A mixture of cereal species that are sown and harvested together. It does not include: meslin (a mixture wheat/rye)." |
| Grapefruit (inc. pomelos) | 507 | Rutaceae | *Citrus maxima* | (Burm. f.) Merr. | FAO also lists *Citrus grandis* (L.) Osbeck, but this is a synonym for *C. maxima* |
| Grapefruit (inc. pomelos) | 507 | Rutaceae | *Citrus* x *paradisi* | Macfad. | FAO lists this as *Citrus paradisi* |
| Grapes | 560 | Vitaceae | *Vitis vinifera* | L. |  |
| Groundnuts, with shell | 242 | Fabaceae | *Arachis hypogaea* | L. |  |
| Hazelnuts, with shell | 225 | Betulaceae | *Corylus avellana* | L. |  |
| Hemp tow waste | 777 | Cannabaceae | *Canabis sativa* | L. |  |
| Hempseed | 336 | Cannabaceae | *Canabis sativa* | L. |  |
| Hops | 677 | Cannabaceae | *Humulus lupulus* | L. |  |
| Jojoba seed | 277 | Simmondsiaceae | *Simmondsia chinensis* | (Link) C. K. Schneid. | FAO also lists the synonym *Simmondsia californica* Nutt. |
| Jute | 780 | Malvaceae | *Corchorus capsularis* | L. |  |
| Jute | 780 | Malvaceae | *Corchorus olitorius* | L. |  |
| Kapok fruit | 310 | Malvaceae | *Ceiba pendantra* | (L.) Gaertn. |  |
| Karite nuts (sheanuts) | 263 | Sapotaceae | *Vitellaria paradoxa* | C.F. Gaertn. | FAO lists the synonym *Butyrospermum parkii* (G. Don) Kotschy |
| Kiwi fruit | 592 | Actinidiaceae | *Actinidia deliciosa* | C.F.Liang & A.R.Ferguson |  |
| Kola nuts | 224 | Malvaceae | *Cola acuminata* | (P. Beauv.) Schott & Endl. |  |
| Kola nuts | 224 | Malvaceae | *Cola nitida* | (Vent.) A. Chev. | FAO also lists the synonym *Cola vera* K.Schum |
| Leeks, other alliaceous vegetables | 407 | Liliaceae | *Allium porrum* | L. |  |
| Leeks, other alliaceous vegetables | 407 | Liliaceae | *Allium schoenoprasum* | L. |  |
| Lemons and limes | 497 | Rutaceae | *Citrus* x *aurantiifolia* | (Christm.) Swingle | FAO lists the synonym *Citrus aurantiifolia* (Christm.) Swingle |
| Lemons and limes | 497 | Rutaceae | *Citrus limetta* | Risso |  |
| Lemons and limes | 497 | Rutaceae | *Citrus* x *limon* | (L.) Burm.f. | FAO lists the synonym *Citrus limon* (L.) Burm. f. |
| Lentils | 201 | Fabaceae | *Lens culinaris* | Medikus | FAO lists synonyms *Lens esculenta* Moench and Ervum lens L. |
| Lettuce and chicory | 372 | Asteraceae | *Cichorium endivia* | L. | FAO lists *Cichorium endivia* var. *crispa* and *Cichorium endivia* var. *latifolia* |
| Lettuce and chicory | 372 | Asteraceae | *Cichorium intybus* | L. | FAO lists synonym *Cichorium intybus* var. *foliosum* |
| Lettuce and chicory | 372 | Asteraceae | *Lactuca sativa* | L. |  |
| Linseed | 333 | Linaceae | *Linum usitatissimum* | L. |  |
| Lupins | 210 | Fabaceae | *Lupinus spp* | NA | FAO does not list any species; USDA recognizes over 50 species in the genus (all of which have common names that include "lupin")' |
| Maize | 56 | Poaceae | *Zea mays* | L. |  |
| Maize, green | 446 | Poaceae | *Zea mays* | L. | FAO lists this as *Zea mays*, particularly var. *saccharata* |
| Mangoes, mangosteens, guavas | 571 | Anacardiaceae | *Mangifera indica* | L. |  |
| Manila fibre (abaca) | 809 | Musaceae | *Musa textilis* | Née |  |
| Mate | 671 | Aquifoliaceae | *Ilex paraguariensis* | A. St. Hil. |  |
| Melons, other (inc.cantaloupes) | 568 | Cucurbitaceae | *Cucumis melo* | L. |  |
| Melonseed | 299 | Cucurbitaceae | *Cucumis melo* | L. |  |
| Millet | 79 | Poaceae | *Echinochloa esculenta* | (A. Braun) H. Scholz | FAO lists this species as "Japanese millet", with the following taxonomy: *Echinocloa frumentacea*. However, this species is not acknowledged by USDA, and *E*. *esculenta* is widely regarded as Japanese millet (although not listed explicitly by FAO, it is included here) |
| Millet | 79 | Poaceae | *Eleusine coracana* | (L.) Gaertn. |  |
| Millet | 79 | Poaceae | *Panicum miliaceum* | L. |  |
| Millet | 79 | Poaceae | *Paspalum scrobiculatum* | L. |  |
| Millet | 79 | Poaceae | *Pennisetum glaucum* | (L.) R. Br. |  |
| Millet | 79 | Poaceae | *Setaria italica* | (L.) P. Beauv. |  |
| Mushrooms and truffles | 449 | Agaricaceae | *Agaricus bisporus* | (J.E.Lange) Imbach |  |
| Mushrooms and truffles | 449 | Agaricaceae | *Agaricus campestris* | L. |  |
| Mushrooms and truffles | 449 | Boletaceae | *Boletus edulis* | Bull. (1782) |  |
| Mushrooms and truffles | 449 | Morchellaceae | *Morchella spp* | (L.) Pers. |  |
| Mushrooms and truffles | 449 | Toberaceae | *Tuber magnatum* | P.Micheli ex F.H.Wigg. |  |
| Mustard seed | 292 | Brassicaceae | *Brassica nigra* | (L.) W.D.J. Koch | FAO also lists the synonym *Sinapis* *nigra* L. |
| Mustard seed | 292 | Brassicaceae | *Sinapis alba* | L. | FAO also lists the synonyms *Brassica alba* Rabenh., non L. and *Brassica hirta* Moench |
| Nutmeg, mace and cardamoms | 702 | Zingiberaceae | *Aframomum angustifolium* | Sonn. | This taxonomy is very confusing; synonym includes *Amomum angustifolium* |
| Nutmeg, mace and cardamoms | 702 | Zingiberaceae | *Aframomum melegueta* | Schum. | FAO also lists in this group *Aframomum hambury*, but information on this species cannot be found |
| Nutmeg, mace and cardamoms | 702 | Zingiberaceae | *Amomum aromaticum* | Roxb. |  |
| Nutmeg, mace and cardamoms | 702 | Zingiberaceae | *Elettaria cardamomum* | (L.) Maton | FAO also lists synonym *Amomum cardamomum* L. |
| Nutmeg, mace and cardamoms | 702 | Myristicaceae | *Myristica fragrans* | Houtt. |  |
| Nuts, nes | 234 | Burseraceae | *Canarium album* | (Lour.) Rausch. | FAO lists this (and other *Canarium* species) as *Canarium* spp. But this species corresponds to the FAO commodity (Chinese olives) |
| Nuts, nes | 234 | Burseraceae | *Canarium indicum* | L. | FAO lists this (and other *Canarium* species) as simply *Canarium* spp. But this species corresponds to the FAO commodity Java almond) |
| Nuts, nes | 234 | Burseraceae | *Canarium ovatum* | Engl. | FAO lists this (and other *Canarium* species) as simply *Canarium* spp. But this species corresponds to the FAO commodity (pili nut) |
| Nuts, nes | 234 | Burseraceae | *Canarium pimela* | Konig | FAO lists this (and other *Canarium* species) as simply *Canarium* spp. But this species corresponds to the FAO commodity (Chinese olives) |
| Nuts, nes | 234 | Juglandaceae | *Carya illinoinensis* | (Wangenh.) K. Koch |  |
| Nuts, nes | 234 | Caryocaraceae | *Caryocar nuciferum* | L. |  |
| Nuts, nes | 234 | Lecythidaceae | *Lecythis zabucajo* | Aubl. |  |
| Nuts, nes | 234 | Proteaceae | *Macadamia ternifolia* | Maiden & Betche |  |
| Nuts, nes | 234 | Pinaceae | *Pinus pinea* | L. |  |
| Oats | 75 | Poaceae | *Avena sativa* | L. | FAO also lists *Avena* spp., but this may correspond to ~ 15 other species as per USDA |
| Oil, palm fruit | 254 | Arecaceae | *Elais guineensis* | Jacq. |  |
| Oilseeds nes | 339 | Euphorbiaceae | *Aleurites moluccanus* | (L.) Willd. | FAO lists the synonym *Aleurites moluccana* (L.) Willd., orth. var. |
| Oilseeds nes | 339 | Arecaceae | *Astrocaryum aculeatum* | G. Mey. | FAO lists this only as *Astrocaryum* spp.; USDA does not recognize this genus, however, FAO lists this item as "tukuma kernel", which corresponds with *A*. *aculeatum* |
| Oilseeds nes | 339 | Meliaceae | *Carapa guianensis* | Aubl. |  |
| Oilseeds nes | 339 | Euphorbiaceae | *Croton tiglium* | L. |  |
| Oilseeds nes | 339 | Fagaceae | *Fagus sylvatica* | L. |  |
| Oilseeds nes | 339 | Asteraceae | *Guizotia abyssinica* | (L. f.) Cass. |  |
| Oilseeds nes | 339 | Euphorbiaceae | *Jatropha curcas* | L. |  |
| Oilseeds nes | 339 | Chrysobalanaceae | *Licania rigida* | Benth. |  |
| Oilseeds nes | 339 | Sapotaceae | *Madhuca indica* | J.F. Gmel. | FAO lists the synonym *Bassia latifolia*Roxb. |
| Oilseeds nes | 339 | Lamiaceae | *Perilla frutescens* | (L.) Britton |  |
| Oilseeds nes | 339 | Fabaceae | *Pongamia glabra* | (L.) Panigrahi | FAO lists the synonym *Pongamia glabra* Vent. |
| Oilseeds nes | 339 | Dipterocarpaceae | *Shorea robusta* | C.F. Gaertn. |  |
| Okra | 430 | Malvaceae | *Abelmoschus esculentus* | (L.) Moench | FAO also lists synonym *Hibiscus esculentus* L. |
| Olives | 260 | Oleaceae | *Olea europaea* | L. |  |
| Onions, dry | 403 | Liliaceae | *Allium cepa* | L. |  |
| Onions, shallots, green | 402 | Liliaceae | *Allium ascalonicum* | L. | While USDA recognizes this taxon, it may be a synonym for *Allium cepa* L. var. *aggregatum* G. Don (as per the US National Germplasm database) |
| Onions, shallots, green | 402 | Liliaceae | *Allium cepa* | L. |  |
| Onions, shallots, green | 402 | Liliaceae | *Allium fistulosum* | L. |  |
| Oranges | 490 | Rutaceae | *Citrus* x *aurantium* | L. | FAO lists this as *Citrus aurantium* |
| Oranges | 490 | Rutaceae | *Citrus* x *sinensis* | (L.) Osbeck | FAO lists this as *Citrus sinensis* |
| Papayas | 600 | Caricaceae | *Carica papaya* | L. |  |
| Peaches and nectarines | 534 | Rosaceae | *Prunus persica* | Batsch | FAO also lists synonym *Amygdalus persica* L. Note however, this is technically a synonym for a variety of *P*. *persica* (var. *persica*); FAO also lists synonym *Persica laevis* |
| Pears | 521 | Rosaceae | *Pyrus communis* | L. |  |
| Peas, dry | 187 | Fabaceae | *Pisum sativum* | L. | FAO also lists synonym *Pisum arvense* L. |
| Peas, green | 417 | Fabaceae | *Pisum sativum* | L. | FAO does not list any synonyms for this group (unlike group 187) |
| Pepper (piper spp.) | 687 | Piperaceae | *Piper longum* | L. |  |
| Pepper (piper spp.) | 687 | Piperaceae | *Piper nigrum* | L. |  |
| Peppermint | 748 | Lamiaceae | *Mentha spicata* | L. | FAO only lists *Metha* spp., however, since this commodity group is called "peppermint, spearmint", this species is added as spearmint as per USDA taxonomy |
| Peppermint | 748 | Lamiaceae | *Mentha* x *piperita* | Willd. |  |
| Persimmons | 587 | Ebenaceae | *Diospyros kaki* | L. f. |  |
| Persimmons | 587 | Ebenaceae | *Diospyros virginiana* | L. |  |
| Pigeon peas | 197 | Fabaceae | *Cajanus cajan* | (L.) Millsp. |  |
| Pineapples | 574 | Bromeliaceae | *Ananas comosus* | (L.) Merr. | FAO also lists synonym *Ananas sativus* |
| Pistachios | 223 | Anacardiaceae | *Pistacia vera* | L. |  |
| Plantains | 489 | Musaceae | *Musa* x *paradisiaca* | L. | FAO lists this as *Musa paradisiaca* |
| Plums and sloes | 536 | Rosaceae | *Prunus domestica* | L. |  |
| Plums and sloes | 536 | Rosaceae | *Prunus spinosa* | L. |  |
| Poppy seed | 296 | Papaveraceae | *Papaver somniferum* | L. |  |
| Potatoes | 116 | Solanaceae | *Solanum tuberosum* | L. |  |
| Pulses, nes | 211 | Fabaceae | *Canavalia ensiformis* | (L.) DC. | FAO lists this as *Canavalia* spp. with the common names "jack or sword bean"; this species refers to "jack bean" as per USDA |
| Pulses, nes | 211 | Fabaceae | *Canavalia gladiata* | (Jacq.) DC. | FAO lists this as *Canavalia* spp. with the common names "jack or sword bean"; this species refers to "sword bean" as per USDA |
| Pulses, nes | 211 | Fabaceae | *Cyamopsis tetragonoloba* | (L.) Taubert |  |
| Pulses, nes | 211 | Fabaceae | *Lablab purpureus* | L. | FAO lists this as *Dolichos* spp. with the common names hyacinth or lablab; this common term seems to refer to this species listed here (which is recognized by USDA, technically as *Lablab purpureus* subsp. *purpureus*) |
| Pulses, nes | 211 | Fabaceae | *Mucuna pruriens* | (L.) DC. | FAO lists this as *Stizolobium* spp. or "velvet bean", which is a synonym for two different varieties of the species listed here |
| Pulses, nes | 211 | Fabaceae | *Pachyrhizus erosus* | (L.) Urb. |  |
| Pulses, nes | 211 | Fabaceae | *Psophocarpus tetragonolobus* | (L.) DC. |  |
| Pumpkins, squash and gourds | 394 | Cucurbitaceae | *Cucurbita pepo* | L. | FAO lists this as *Cucurbita* spp. but all major cultivated pumpkins, squahses and gourds are varieties of this species |
| Pyrethrum, dried | 754 | Asteraceae | *Chrysanthemum cinerariifolium* | (Trevir.) Vis. |  |
| Quinces | 523 | Rosaceae | *Chaenomeles japonica* | (Thunb.) Lindl. ex Spach | FAO lists synonym *Cydonia japonica* (Thunb.) Pers. |
| Quinces | 523 | Rosaceae | *Cydonia oblonga* | Mill. | FAO also lists synonym *Cydonia vulgaris* Pers. |
| Quinoa | 92 | Amaranthaceae | *Chenopodium quinoa* | Willd. |  |
| Ramie | 788 | Urticaceae | *Boehmeria nivea* | (L.) Gaudich. | FAO also lists synonym/ possibly subspecies *Boehmeria tenacissima* Gaudich. |
| Rapeseed | 270 | Brassicaceae | *Brassica napus* | L. |  |
| Raspberries | 547 | Rosaceae | *Rubus idaeus* | L. |  |
| Rice, paddy | 27 | Poaceae | *Oryza glaberrima* | Steud. | FAO lists this simply as *Oryza* spp., however, this is the only other cultivated rice species |
| Rice, paddy | 27 | Poaceae | *Oryza sativa* | L. |  |
| Roots and tubers, nes | 149 | Apiaceae | *Arracacia xanthorrhiza* | E.N. Bancroft | FAO lists this as *Arracacoa xanthorrhiza* but this is a misspelling |
| Roots and tubers, nes | 149 | Cyperaceae | *Cyperus esculentus* | L. |  |
| Roots and tubers, nes | 149 | Asteraceae | *Helianthus tuberosus* | L. |  |
| Roots and tubers, nes | 149 | Marantaceae | *Maranta arundinacea* | L. |  |
| Roots and tubers, nes | 149 | Arecaceae | *Metroxylon sagu* | Rottb. | FAO only lists *Metroxylon* spp. but the corresponding commodity is sago palm, which corresponds with this species (as per USDA) |
| Roots and tubers, nes | 149 | Oxalidaceae | *Oxalis tuberosa* | Molina |  |
| Roots and tubers, nes | 149 | Fabaceae | *Pachyrhizus erosus* | (L.) Urb. | FAO also lists the synonym *Pachyrhizus angulatus* Rich. ex DC. |
| Roots and tubers, nes | 149 | Tropaeolaceae | *Tropaeolum tuberosum* | Ruiz & Pav. |  |
| Roots and tubers, nes | 149 | Basellaceae | *Ullucus tuberosus* | Loz. |  |
| Rubber, natural | 836 | Euphorbiaceae | *Hevea brasiliensis* | (Willd. ex A. Juss.) Müll. Arg. |  |
| Rye | 71 | Poaceae | *Secale cereale* | L. |  |
| Safflower seed | 280 | Asteraceae | *Carthamus tinctorius* | L. |  |
| Seed cotton | 328 | Malvaceae | *Gossypium arboreum* | L. | FAO lists only *Gossypium* spp.; note, *G*. *herbaceum* is cited as a cultivated cotton species, but USDA recognizes this as a synonym for a variety of *G*. *hirsutum* |
| Seed cotton | 328 | Malvaceae | *Gossypium barbadense* | L. | FAO lists only *Gossypium* spp.; note, *G*. *herbaceum* is cited as a cultivated cotton species, but USDA recognizes this as a synonym for a variety of *G*. *hirsutum* |
| Seed cotton | 328 | Malvaceae | *Gossypium hirsutum* | L. | FAO lists only *Gossypium* spp.; note, *G*. *herbaceum* is cited as a cultivated cotton species, but USDA recognizes this as a synonym for a variety of *G*. *hirsutum* |
| Sesame seed | 289 | Pedaliaceae | *Sesamum orientale* | L. | FAO lists synonym *Sesamum indicum* L. |
| Sisal | 789 | Asparagaceae | *Agave sisalana* | Perrine |  |
| Sorghum | 83 | Poaceae | *Sorghum bicolor* | (L.) Moench | FAO lists *S*. *guineense*, *S*. *vulgare*, and *S*. *dura*, but these are synonyms for subspecies of *S*. *bicolor* (as per USDA) |
| Soybeans | 236 | Fabaceae | *Glycine max* | (L.) Merr. | FAO lists synonym *Glycine soja* (L.) Merr., non Siebold & Zucc., nom. illeg. |
| Spices, nes | 723 | Apiaceae | *Anethum graveolens* | L. |  |
| Spices, nes | 723 | Iridaceae | *Crocus sativus* | L. |  |
| Spices, nes | 723 | Zingiberaceae | *Curcuma longa* | L. |  |
| Spices, nes | 723 | Lauraceae | *Laurus nobilis* | L. |  |
| Spices, nes | 723 | Lamiaceae | *Thymus vulgaris* | L. |  |
| Spices, nes | 723 | Fabaceae | *Trigonella foenum graecum* | L. |  |
| Spinach | 373 | Amaranthaceae | *Spinacia oleracea* | L. |  |
| Strawberries | 544 | Rosaceae | *Fragaria* x *ananassa* | Duchesne | FAO lists this only as *Fragaria* spp. |
| String beans | 423 | Fabaceae | *Phaseolus vulgaris* | L. |  |
| Sugar beet | 157 | Amaranthaceae | *Beta vulgaris* | L. | FAO lists the variety *Beta vulgaris* var. *altissima* |
| Sugar cane | 156 | Poaceae | *Saccharum officinarum* | L. |  |
| Sugar crops, nes | 161 | Sapindaceae | *Acer saccharum* | Marshall |  |
| Sugar crops, nes | 161 | Arecaceae | *Arenga saccharifera* | (Wurmb) Merr. |  |
| Sugar crops, nes | 161 | Poaceae | *Sorghum bicolor* | (L.) Moench | FAO lists *Sorghum saccharatum* (L.) Moench, nom. utique rej., which is a synonym for the subspecies *Sorghum bicolor* (L.) Moench subsp. Bicolor |
| Sunflower seed | 267 | Asteraceae | *Helianthus annuus* | L. |  |
| Sweet potatoes | 122 | Convolvulaceae | *Ipomoea batatas* | (L.) Lam. |  |
| Tallowtree seed | 305 | Dipterocarpaceae | *Shorea aptera* | Burck | This name appears to be unresolved; synonym includes *S*. *palembanica* Miq. which is also unresolved |
| Tallowtree seed | 305 | Dipterocarpaceae | *Shorea stenocarpa* | unknown | This name does not appear in any taxonomic databases |
| Tallowtree seed | 305 | Euphorbiaceae | *Triadica sebifera* | (L.) Small | FAO lists the synonym *Sapium sebiferum* (L.) Roxb. and *Stillingia sebifera* |
| Tangerines, mandarins, clementines, satsumas | 495 | Rutaceae | *Citrus reticulata* | Blanco | FAO also lists *Citrus unshiu*, however the USDA recognizes this as a subspecies of *C*. *reticulata* |
| Taro (cocoyam) | 136 | Araceae | *Colocasia esculenta* | (L.) Schott |  |
| Tea | 667 | Theaceae | *Camellia sinensis* | (L.) Kuntze | FAO also lists *Thea sinensis* L. and *Thea assamica* J. W. Masters; but the USDA recognizes these are both synonyms for varieties of *Camellia sinensis* (L.) Kuntze (var. *sinensis*). |
| Tobacco, unmanufactured | 826 | Solanaceae | *Nicotiana tabacum* | L. |  |
| Tomatoes | 388 | Solanaceae | *Solanum lycopersicum* | L. | FAO lists *Lycopersicon esculentum* Mill. Which is a synonym for a variety of *S*. *lycopersicon* |
| Triticale | 97 | Poaceae | X *Triticosecale* | Wittm. ex A. Camus. | FAO does not list any taxa; this is a hybrid at the genus level with no species identity |
| Tung nuts | 275 | Euphorbiaceae | *Vernicia fordii* | (Hemsl.) Airy Shaw | FAO lists synonym *Aleurites fordii* Hemsl. |
| Tung nuts | 275 | Euphorbiaceae | *Vernicia montana* | Lour. | FAO lists synonym *Aleurites cordata* auct. non (Thunb.) R. Br. ex Steud., orth. var. |
| Vanilla | 692 | Orchidaceae | *Vanilla planifolia* | Jacks. |  |
| Vanilla | 692 | Orchidaceae | *Vanilla pompona* | Schiede |  |
| Vegetables, fresh nes | 463 | Apiaceae | *Anthriscus cerefolium* | (L.) Hoffm. |  |
| Vegetables, fresh nes | 463 | Apiaceae | *Apium graveolens* | L. |  |
| Vegetables, fresh nes | 463 | Asteraceae | *Artemisia dracunculus* | L. |  |
| Vegetables, fresh nes | 463 | Poaceae | *Bambusa blumeana* | Schult. f. | FAO only lists this as *Bambusa* spp.; but these are the most common harvested species |
| Vegetables, fresh nes | 463 | Poaceae | *Bambusa odashimae* | Hatus. ex D.Z.Li & Stapleton | FAO only lists this as *Bambusa* spp.; but these are the most common harvested species |
| Vegetables, fresh nes | 463 | Poaceae | *Bambusa oldhamii* | W. Munro [excluded] | FAO only lists this as *Bambusa* spp.; but these are the most common harvested species |
| Vegetables, fresh nes | 463 | Amaranthaceae | *Beta vulgaris* | L. |  |
| Vegetables, fresh nes | 463 | Brassicaceae | *Brassica oleracea* | L. | FAO cites this as *B*. *napus*, but then ascribes this as ruttabagga/ swedes; however *B*. *napus* is rape seed |
| Vegetables, fresh nes | 463 | Capparaceae | *Capparis spinosa* | L. |  |
| Vegetables, fresh nes | 463 | Brassicaceae | *Cochlearia armoracia* | G. Gaertn., B. Mey. & Scherb. |  |
| Vegetables, fresh nes | 463 | Asteraceae | *Cynara cardunculus* | L. |  |
| Vegetables, fresh nes | 463 | Poaceae | *Fargesia spathacea* | Franch. | FAO only lists this as *Bambusa* spp.; but these are the most common harvested species |
| Vegetables, fresh nes | 463 | Apiaceae | *Foeniculum vulgare* | Mill. |  |
| Vegetables, fresh nes | 463 | Brassicaceae | *Lepidium sativum* | L. |  |
| Vegetables, fresh nes | 463 | Lamiaceae | *Majorana hortensis* | L. |  |
| Vegetables, fresh nes | 463 | Brassicaceae | *Nasturtium officinale* | W.T. Aiton |  |
| Vegetables, fresh nes | 463 | Apiaceae | *Pastinaca sativa* | L. |  |
| Vegetables, fresh nes | 463 | Apiaceae | *Petroselinum crispum* | (Mill.) Nyman ex A.W. Hill |  |
| Vegetables, fresh nes | 463 | Poaceae | *Phyllostachys bambusoides* | Siebold & Zucc. | FAO only lists this as *Bambusa* spp.; but these are the most common harvested species |
| Vegetables, fresh nes | 463 | Poaceae | *Phyllostachys edulis* | (Carrière) J. Houz. | FAO only lists this as *Bambusa* spp.; but these are the most common harvested species |
| Vegetables, fresh nes | 463 | Brassicaceae | *Raphanus sativu* | L. |  |
| Vegetables, fresh nes | 463 | Polygonaceae | *Rheum rhabarbarum* | L. | FAO lists only *Rheum* spp., and notes it represents rhubarb |
| Vegetables, fresh nes | 463 | Polygonaceae | *Rumex acetosa* | L. |  |
| Vegetables, fresh nes | 463 | Lamiaceae | *Satureja hortensis* | L. |  |
| Vegetables, fresh nes | 463 | Asteraceae | *Scorzonera hispanica* | L. |  |
| Vegetables, fresh nes | 463 | Poaceae | *Sinocalamus latiflorus* | (Munro) McClure | FAO only lists this as *Bambusa* spp.; but these are the most common harvested species |
| Vegetables, fresh nes | 463 | Asteraceae | *Tragopogon porrifolius* | L. |  |
| Vegetables, leguminous nes | 420 | Fabaceae | *Vicia faba* | L. |  |
| Vetches | 205 | Fabaceae | *Vicia sativa* | L. |  |
| Walnuts, with shell | 222 | Juglandaceae | *Juglans regia* | L. | FAO has this misspelled as *Juglands regia* |
| Watermelons | 567 | Cucurbitaceae | *Citrullus lanatus* | (Thunb.) Matsum. & Nakai | FAO lists *Citrullus vulgaris* Schrad. which is a synonym for a subspecies of *C*. *lanatus* |
| Wheat | 15 | Poaceae | *Triticum aestivum* | L. |  |
| Wheat | 15 | Poaceae | *Triticum durum* | Desf. |  |
| Wheat | 15 | Poaceae | *Triticum spelta* | L. |  |
| Yams | 137 | Dioscoreaceae | *Dioscorea alata* | L. | FAO lists only *Dioscorea* spp. but the species below are those identified as the most important commercially cultivated species, which are then cross-checked with USDA. |
| Yams | 137 | Dioscoreaceae | *Dioscorea bulbifera* | L. |  |
| Yams | 137 | Dioscoreaceae | *Dioscorea cayenensis* | Lam. |  |
| Yams | 137 | Dioscoreaceae | *Dioscorea dumetorum* | (Kunth) Pax |  |
| Yams | 137 | Dioscoreaceae | *Dioscorea esculenta* | (Lour.) Burkill |  |
| Yams | 137 | Dioscoreaceae | *Dioscorea polystachya* | Turcz. | USDA lists this as the synonym *Dioscorea oppositifolia* L. |
| Yams | 137 | Dioscoreaceae | *Dioscorea rotundata* | Poir. |  |
| Yams | 137 | Dioscoreaceae | *Dioscorea trifida* | L. f. |  |
| Yautia (cocoyam) | 135 | Araceae | *Xanthosoma sagittifolium* | (L.) Schott | FAO lists *Xanthosoma* spp. |

**Table B. Model diagnostics (Akaike’s Information Criterion (AIC)) for six different models used to describe change in four different diversity metrics and total agricultural area, as a function of year.** Location names follow those of the Food and Agricultural Organization of the United Nations, and in all models year ranged from 1961-2014 (*n*=54) except for Central Asia where data was available only from 1992-2014 (*n*=23 years). Models that failed to converge are denoted by “NA” and the most parsimonious model fits as per the lowest AIC value are highlighted in bold.

|  |  |  | **Taxonomic diversity** | | **Phylogenetic diversity** | |
| --- | --- | --- | --- | --- | --- | --- |
| **Location** | **Model** | **Area** | **Richness** | **Simpson** | **PD** | **Rao's D** |
| World | Linear | 1345.466 | 122.3177 | -480.2427 | 556.3278 | 179.4435 |
|  | Polynomial | 1314.59 | 74.16817 | -499.1422 | 523.2779 | 151.8036 |
|  | Unimodal | 1821.755 | 704.8262 | -69.18095 | 1133.796 | 646.3373 |
|  | Asymptotic | 1324.396 | 88.79098 | NA | 533.8651 | NA |
|  | Logistic | 1280.662 | 20.93054 | -525.4173 | 448.1496 | 98.26607 |
|  | Piecewise | **1181.767** | **4.009948** | **-556.7317** | **410.1008** | **94.13431** |
| Australia & New Zealand | Linear | 1202.572 | 239.8951 | -151.3694 | 669.6706 | 359.7629 |
|  | Polynomial | 1089.726 | 211.5455 | -150.2388 | 622.0823 | 357.9147 |
|  | Unimodal | 1573.04 | 637.6976 | -149.2572 | 1074.097 | 569.4114 |
|  | Asymptotic | NA | 216.2011 | -150.1917 | 632.4889 | 358.7083 |
|  | Logistic | 1110.447 | 161.5662 | -151.9774 | 549.9757 | 326.3305 |
|  | Piecewise | **1064.015** | **148.2212** | **-194.7759** | **530.754** | **321.3698** |
| Caribbean | Linear | 844.3429 | 278.8463 | -281.1127 | 684.4831 | 270.8911 |
|  | Polynomial | 718.407 | 280.7478 | -320.8725 | 686.477 | 244.5445 |
|  | Unimodal | 1169.69 | 618.7691 | -111.3899 | 1070.487 | 659.2981 |
|  | Asymptotic | NA | NA | NA | NA | NA |
|  | Logistic | 746.6555 | 187.1611 | -334.4609 | NA | 241.6452 |
|  | Piecewise | **690.3592** | **114.2828** | **-336.9702** | **502.3856** | **228.4554** |
| Central America | Linear | 978.4701 | 334.8624 | -282.8488 | 739.2479 | 216.0285 |
|  | Polynomial | 974.1619 | 320.5255 | -293.8365 | 715.8016 | 215.5283 |
|  | Unimodal | 1423.536 | 663.9389 | -292.848 | 1102.011 | 639.1639 |
|  | Asymptotic | 976.0586 | 325.3649 | -301.3164 | 723.4425 | NA |
|  | Logistic | **807.7927** | **212.6376** | NA | **613.5848** | NA |
|  | Piecewise | 822.1288 | 218.5863 | **-338.617** | 624.6468 | **209.6095** |
| Central Asia | Linear | 433.7092 | 75.96936 | -78.15852 | 230.4658 | **104.2811** |
|  | Polynomial | **408.2076** | 66.27987 | -128.655 | 222.513 | 104.4153 |
|  | Unimodal | 652.2658 | 276.5245 | -74.03965 | 460.8114 | 259.7465 |
|  | Asymptotic | NA | 69.16853 | NA | 224.9777 | NA |
|  | Logistic | 421.0797 | 35.6017 | -102.2086 | 189.4339 | NA |
|  | Piecewise | 1083.58 | **31.61316** | **-338.617** | **188.7149** | 209.6095 |
| East Africa | Linear | 1160.907 | 237.3715 | -441.6705 | 623.7016 | 164.6216 |
|  | Polynomial | 1148.167 | 238.778 | -449.2972 | 625.1678 | 160.0307 |
|  | Unimodal | 1522.17 | 659.2657 | -67.1671 | 1098.362 | 661.0127 |
|  | Asymptotic | NA | NA | NA | 625.2617 | 159.7911 |
|  | Logistic | NA | 127.4654 | NA | 594.6994 | NA |
|  | Piecewise | **1083.58** | **114.0657** | **-459.3618** | **541.4684** | **153.0674** |
| East Asia | Linear | 1248.427 | 312.5695 | -327.3297 | 737.6118 | 285.1497 |
|  | Polynomial | 1195.091 | 312.0577 | -353.9436 | 738.7172 | 273.6669 |
|  | Unimodal | 1598.061 | 670.8301 | -102.8316 | 1106.063 | 647.2902 |
|  | Asymptotic | 1214.977 | 312.7814 | NA | 738.976 | NA |
|  | Logistic | 1155.562 | 195.4718 | -363.7188 | 603.6463 | NA |
|  | Piecewise | **1090.793** | **114.0657** | **-472.6561** | **541.4684** | **164.4864** |
| Eastern Europe | Linear | 1370.58 | 231.0443 | -314.3685 | 638.0742 | 263.1003 |
|  | Polynomial | 1364.584 | 232.277 | -322.7895 | 639.7659 | 236.5828 |
|  | Unimodal | 1579.189 | 641.4425 | -226.4748 | 1073.793 | 624.8085 |
|  | Asymptotic | NA | NA | NA | NA | NA |
|  | Logistic | NA | 175.1541 | -338.7932 | NA | NA |
|  | Piecewise | **945.8686** | **146.8652** | **-345.6732** | **541.4684** | **230.4664** |
| Melanesia | Linear | 537.8616 | 158.8263 | -297.1614 | 568.1824 | 241.646 |
|  | Polynomial | 508.2251 | 160.7952 | -329.4377 | 568.4678 | 221.243 |
|  | Unimodal | 963.4421 | 573.0109 | -185.3464 | 1041.043 | 663.1257 |
|  | Asymptotic | 509.8607 | NA | -339.2007 | 568.7714 | 203.8461 |
|  | Logistic | 501.8006 | 101.7635 | NA | **531.4652** | NA |
|  | Piecewise | **498.7438** | **94.52321** | **-345.6732** | 533.5392 | **182.6827** |
| Micronesia | Linear | 220.2687 | 194.4132 | -247.6374 | 646.757 | 272.6997 |
|  | Polynomial | 222.24 | 196.1458 | -273.3688 | 648.6427 | 250.929 |
|  | Unimodal | 660.0976 | 464.7411 | -212.5076 | 976.1269 | 491.3615 |
|  | Asymptotic | 222.2338 | NA | NA | 648.6104 | NA |
|  | Logistic | NA | NA | NA | NA | NA |
|  | Piecewise | **193.3684** | **94.52321** | **-299.0031** | **533.5392** | **222.2601** |
| Middle Africa | Linear | 857.1184 | 306.1448 | -433.8123 | 693.9473 | 132.1817 |
|  | Polynomial | 855.6389 | 307.4317 | -464.3722 | 695.9056 | 119.665 |
|  | Unimodal | 1456.492 | 623.6603 | -82.17782 | 1072.873 | 668.2393 |
|  | Asymptotic | NA | NA | NA | 695.9169 | NA |
|  | Logistic | NA | 176.3175 | -455.7728 | 587.6933 | NA |
|  | Piecewise | **740.9997** | **173.3601** | **-473.7575** | **578.9647** | **92.47589** |
| North Africa | Linear | 1209.248 | 246.9506 | -352.738 | 652.8535 | 269.2809 |
|  | Polynomial | 1192.426 | 241.6535 | -364.5894 | 649.7273 | 270.3907 |
|  | Unimodal | 1488.423 | 654.2686 | -110.4989 | 1089.942 | 639.9285 |
|  | Asymptotic | NA | 243.5252 | NA | 651.1915 | NA |
|  | Logistic | 1207.25 | 147.4759 | NA | 533.8961 | NA |
|  | Piecewise | **1078.539** | **110.7796** | **-370.9888** | **521.1934** | **267.1433** |
| North America | Linear | 1019.663 | 208.4723 | -350.3919 | 603.0634 | 251.7229 |
|  | Polynomial | 1018.098 | 209.9726 | -351.2692 | 586.7717 | 250.1352 |
|  | Unimodal | 1576.794 | 641.8429 | -256.0272 | 1079.482 | 632.3003 |
|  | Asymptotic | NA | NA | -352.2473 | NA | **250.1006** |
|  | Logistic | NA | 136.222 | NA | **549.7786** | NA |
|  | Piecewise | **976.7688** | **128.2728** | **-366.0192** | 550.3802 | 250.245 |
| Northern Europe | Linear | 943.661 | 290.2353 | -218.6448 | 680.6975 | 307.0661 |
|  | Polynomial | **935.659** | 264.68 | -218.1112 | 669.7617 | 309.0568 |
|  | Unimodal | 1298.805 | 584.8634 | -211.3838 | 1033.055 | 597.5988 |
|  | Asymptotic | NA | NA | NA | NA | 309.0604 |
|  | Logistic | NA | 207.6098 | -257.708 | 634.9262 | 237.0082 |
|  | Piecewise | 985.2874 | **128.2728** | **-317.7394** | **601.9099** | **217.7024** |
| Polynesia | Linear | 421.6506 | 77.42402 | -247.5286 | 515.0642 | 278.4153 |
|  | Polynomial | **372.6124** | 74.17348 | -253.9553 | 473.2956 | 274.9272 |
|  | Unimodal | 704.7574 | 563.9404 | -219.8542 | 1041.829 | 632.0183 |
|  | Asymptotic | NA | NA | NA | 463.0698 | NA |
|  | Logistic | 390.2574 | 73.84924 | NA | NA | NA |
|  | Piecewise | 985.2874 | **69.62815** | **-262.005** | **369.8527** | **217.7024** |
| South America | Linear | 1104.077 | 207.7129 | -263.7503 | 655.4504 | 255.5736 |
|  | Polynomial | 1056.658 | 209.435 | -373.5172 | 629.9108 | 198.492 |
|  | Unimodal | 1587.467 | 679.0487 | -106.6761 | 1113.278 | 655.3411 |
|  | Asymptotic | 1049.098 | 209.4844 | NA | 636.3868 | NA |
|  | Logistic | NA | 141.0735 | -404.2486 | 569.2433 | 243.4813 |
|  | Piecewise | **985.2874** | **134.4511** | **-412.1973** | **564.451** | **167.0513** |
| South East Asia | Linear | 1016.474 | 195.1441 | -342.1926 | 576.043 | 161.5965 |
|  | Polynomial | 973.3031 | 197.1381 | -399.0612 | 568.2398 | 147.5545 |
|  | Unimodal | 1408.878 | 652.8093 | -396.3054 | 1095.126 | 641.0013 |
|  | Asymptotic | NA | 197.14 | -400.7841 | 570.827 | 147.9694 |
|  | Logistic | NA | **66.30488** | -400.8176 | **506.4786** | 148.8386 |
|  | Piecewise | **961.4959** | 68.76215 | **-419.7139** | 564.451 | **146.3938** |
| Southern Africa | Linear | 961.436 | 142.5415 | -255.0264 | 554.2004 | 322.885 |
|  | Polynomial | 939.8142 | 125.2855 | **-261.778** | 543.2789 | 280.9837 |
|  | Unimodal | 1458.51 | 618.8443 | -237.7834 | 1059.473 | 599.2002 |
|  | Asymptotic | NA | NA | NA | NA | NA |
|  | Logistic | 952.4445 | 94.95267 | NA | 505.0313 | **283.3707** |
|  | Piecewise | **850.999** | **86.22187** | -260.8101 | **497.2977** | 284.1809 |
| Southern Asia | Linear | 1106.737 | 203.0575 | -460.4403 | 591.7576 | 207.8688 |
|  | Polynomial | **1049.213** | 203.5804 | -499.5673 | 592.1265 | 163.9187 |
|  | Unimodal | 1533.271 | 665.0553 | -103.2208 | 1101.415 | 642.7744 |
|  | Asymptotic | NA | NA | NA | 592.4763 | NA |
|  | Logistic | NA | 75.42718 | NA | NA | NA |
|  | Piecewise | NA | **65.60883** | **-508.9241** | **497.2977** | **142.5307** |
| Southern Europe | Linear | 925.6803 | 234.2225 | -367.4151 | 666.1152 | 227.8793 |
|  | Polynomial | 876.6464 | 235.7635 | -413.5983 | 668.0556 | **220.0415** |
|  | Unimodal | 1372.595 | 663.9582 | -108.5884 | 1097.101 | 655.3497 |
|  | Asymptotic | NA | 235.9097 | -399.3751 | 668.0782 | 223.1096 |
|  | Logistic | NA | 129.4021 | -446.128 | 562.8463 | 145.1045 |
|  | Piecewise | **841.8708** | **125.2611** | **-454.6561** | **497.2977** | 128.8291 |
| West Africa | Linear | 1142.187 | 270.1243 | -408.5243 | 640.3535 | 254.1234 |
|  | Polynomial | 1092.297 | 270.7536 | -410.3305 | 642.1079 | 246.606 |
|  | Unimodal | 1503.447 | 629.7268 | -80.13617 | 1074.877 | 660.7477 |
|  | Asymptotic | NA | NA | NA | NA | NA |
|  | Logistic | 1046.284 | 131.892 | NA | 571.2995 | NA |
|  | Piecewise | **1013.557** | **115.3886** | **-445.8689** | **533.4503** | **175.573** |
| Western Asia | Linear | 1223.846 | 165.8084 | -344.3923 | 612.6462 | 214.3691 |
|  | Polynomial | 1223.003 | 166.9017 | -350.1115 | 603.3409 | 212.1926 |
|  | Unimodal | 1492.014 | 661.9033 | -325.2628 | 1097.27 | 632.268 |
|  | Asymptotic | NA | NA | NA | NA | 211.9823 |
|  | Logistic | **1095.326** | 149.1935 | NA | 573.5674 | NA |
|  | Piecewise | 1097.126 | **132.2582** | **-359.146** | **571.3382** | **204.032** |
| Western Europe | Linear | 819.1609 | 203.9264 | -355.863 | 631.1681 | 294.2879 |
|  | Polynomial | 756.1532 | 204.6747 | -355.9978 | 630.6749 | 296.17 |
|  | Unimodal | 1344.989 | 634.539 | -192.2369 | 1066.163 | 631.3731 |
|  | Asymptotic | 756.6929 | 204.932 | NA | 631.1572 | NA |
|  | Logistic | 758.1648 | 168.9053 | NA | NA | 251.827 |
|  | Piecewise | **746.3607** | **130.424** | **-422.125** | **583.786** | **172.2661** |

**Table C. Model parameters for piecewise linear regressions describing changes in crop diversity and agricultural area through time in 22 regions.** Model functional form followed Equations 2 and 3 in the main text, and acronyms for diversity indices are as follows: QE_phy_=phylogenetic Rao’s quadratic entropy, and Ag. Area=agricultural area. Location names follow those of the Food and Agricultural Organization of the United Nations. For each model, year ranged from 1961-2014 (*n*=54) except for Central Asia where data was available only from 1992-2014 (*n*=23 years). Model parameters and associated confidence limits surrounding them (in parentheses) were generated through bootstrapping with replacement (with *n*=500 randomizations performed).

| **Metric** | **Region** | ***r*^2^** | **Intercept** | **Slope 1** | **Slope 2** | **Slope 3** | **ψ1** | **ψ2** |
| --- | --- | --- | --- | --- | --- | --- | --- | --- |
| Species richness | World | 0.988 | 151  (86.8) | 0.0001  (0.00001) | 0.2  (0.001) | -0.2  (0.001) | 1967.9  (1967.5, 1968.4) | 1991.9  (1991.4, 1992.4) |
| - | Eastern Africa | 0.992 | -153  (42.9) | 0.1  (0.001) | 0.9  (0.1) | -0.9  (0.1) | 1982.8  (1982.6, 1983) | 1994.9  (1994.7, 1995.1) |
| - | Middle Africa | 0.989 | 78.5  (74.4) | -0.01  (0.001) | 2.1  (0.1) | -1.9  (0.1) | 1982.7  (1982.6, 1982.8) | 1991.4  (1991.3, 1991.4) |
| - | Northern Africa | 0.993 | -447.1  (39.0) | 0.3  (0.001) | 1.4  (0.2) | -1.6  (0.2) | 1983.6  (1983.5, 1983.7) | 1989.9  (1989.8, 1990) |
| - | Southern Africa | 0.972 | 67.0  (48.8) | 0.00001  (0.000001) | 0.3  (0.001) | -0.3  (0.1) | 1975.6  (1975.5, 1975.8) | 1995.4  (1995.3, 1995.6) |
| - | West Africa | 0.994 | -109.5  (38.2) | 0.1  (0.001) | 1.8  (0.1) | -1.7  (0.1) | 1984.8  (1984.7, 1984.9) | 1992.3  (1992.2, 1992.4) |
| - | Caribbean | 0.993 | 173.0  (40.5) | -0.1  (0.001) | 10.5  (0.9) | -10.2  (0.9) | 1984  (1983.9, 1984.1) | 1985.7  (1985.6, 1985.8) |
| - | Central America | 0.985 | -267.7  (182.3) | 0.2  (0.1) | 2.1  (0.2) | -2.2  (0.1) | 1977.2  (1977.1, 1977.3) | 1986.8  (1986.7, 1986.9) |
| - | North America | 0.985 | 114.3  (96.5) | -0.02  (0.1) | 0.5  (0.1) | -0.6  (0.1) | 1975.9  (1975.7, 1976) | 1992.8  (1992.6, 1993) |
| - | South America | 0.991 | -498.4  (51.9) | 0.3  (0.001) | 0.7  (0.1) | -0.8  (0.1) | 1981.4  (1981, 1981.8) | 1992.3  (1991.9, 1992.7) |
| - | Central Asia | 0.979 | -299.6  (127.0) | 0.2  (0.1) | 1.8  (0.6) | -2.0  (0.6) | 1999.1  (1999.1, 1999.1) | 2001.2  (2001.2, 2001.2) |
| - | East Asia | 0.991 | -104.8  (63.4) | 0.1  (0.001) | 8.0  (1.4) | -7.9  (1.4) | 1983.5  (1983.5, 1983.5) | 1985.7  (1985.7, 1985.7) |
| - | South-east Asia | 0.985 | -8.5  (21.9) | 0.1  (0.001) | 1.4  (0.2) | -1.5  (0.2) | 1985.3  (1985.1, 1985.4) | 1991.2  (1991.1, 1991.3) |
| - | South Asia | 0.989 | -18.0  (19.1) | 0.1  (0.001) | 4  (0.6) | -4.0  (0.6) | 1987  (1986.9, 1987.1) | 1991.1  (1991, 1991.2) |
| - | Western Asia | 0.980 | 16.1  (90.2) | 0.04  (0.001) | 0.8  (0.2) | -0.5  (0.2) | 1975.7  (1975.5, 1975.9) | 1981  (1980.8, 1981.2) |
| - | Eastern Europe | 0.982 | -23.2  (62.4) | 0.1  (0.001) | 1.1  (0.1) | -1.0  (0.1) | 1981.9  (1981.8, 1982) | 1989.7  (1989.6, 1989.8) |
| - | Northern Europe | 0.982 | 130.5  (44.3) | -0.04  (0.001) | 4.5  (0.7) | -4.1  (0.7) | 1989.8  (1989.8, 1989.8) | 1992.3  (1992.3, 1992.3) |
| - | Southern Europe | 0.979 | 272.1  (59.3) | -0.1  (0.001) | 1.2  (0.1) | -1.0  (0.1) | 1979.5  (1979.4, 1979.7) | 1988.2  (1988.1, 1988.4) |
| - | Western Europe | 0.977 | -173.7  (46.8) | 0.1  (0.001) | 1.4  (0.2) | -1.4  (0.2) | 1983.8  (1983.7, 1983.9) | 1988  (1987.9, 1988.1) |
| - | Aust. & New Zeal. | 0.957 | -951.7  (45.4) | 0.5  (0.001) | -0.9  (0.2) | 0.6  (0.2) | 1976.7  (1976.4, 1977) | 1985.6  (1985.3, 1985.9) |
| - | Melanesia | 0.977 | 42.2  (57.8) | 0.0001  (0.0001) | 0.4  (0.001) | -0.3  (0.001) | 1976.3  (1976.2, 1976.4) | 1995  (1994.9, 1995.2) |
| - | Micronesia | 0.396 | -164.3  (60.3) | 0.1  (0.001) | -0.8  (0.4) | 0.8  (0.4) | 1986.9  (1986.9, 1986.9) | 1991.7  (1991.7, 1991.7) |
| - | Polynesia | 0.875 | -105  (38.4) | 0.1  (0.001) | -0.1  (0.1) | 0.1  (0.001) | 1978.4  (1978.2, 1978.7) | 1994  (1993.7, 1994.3) |
| Phylogenetic diversity | World | 0.990 | -1111.1  (768.0) | 4.6  (0.4) | 24.8  (3.2) | -29.2  (3.2) | 1980.6  (1980.4, 1980.8) | 1985.5  (1985.3, 1985.7) |
| - | Eastern Africa | 0.988 | -18960.9  (1572.7) | 12.4  (0.8) | 61.6  (10.8) | -68.6  (10.8) | 1988.2  (1987.7, 1988.8) | 1992.7  (1992.2, 1993.2) |
| - | Middle Africa | 0.989 | -8281.5  (3179.8) | 6.3  (1.6) | 75.7  (6.4) | -74.5  (6.4) | 1982.6  (1982.5, 1982.7) | 1991  (1990.9, 1991.1) |
| - | Northern Africa | 0.990 | -7455.9  (1863.0) | 6.4  (0.9) | 68.5  (6.8) | -72.1  (6.8) | 1983.3  (1983.2, 1983.4) | 1988.2  (1988.1, 1988.3) |
| - | Southern Africa | 0.968 | 3978.8  (2016.1) | -0.005  (1.0) | 11.4  (1.2) | -11.4  (2.2) | 1978.7  (1978.7, 1978.7) | 2003.8  (2003.8, 2003.8) |
| - | West Africa | 0.987 | -7874.8  (1723.2) | 6.2  (0.9) | 57.8  (6.0) | -59.7  (6.0) | 1986  (1985.9, 1986.1) | 1992.8  (1992.7, 1992.9) |
| - | Caribbean | 0.996 | 4181.3  (1464.3) | -0.01  (0.7) | 467.3  (33.4) | -457.9  (33.4) | 1984  (1984, 1984) | 1985.1  (1985.1, 1985.1) |
| - | Central America | 0.985 | -26024.1  (7820.6) | 15.9  (4.0) | 78  (7.3) | -90.3  (6.4) | 1977.5  (1977.4, 1977.7) | 1986.6  (1986.5, 1986.8) |
| - | North America | 0.965 | -1574.2  (2283.3) | 3.2  (1.2) | 16.4  (1.8) | -19.6  (4.3) | 1983.9  (1983.9, 1983.9) | 2004.3  (2004.3, 2004.3) |
| - | South America | 0.988 | -15843.1  (7865.5) | 11.2  (4.0) | 23.7  (4.2) | -30.8  (2.1) | 1970.6  (1970.2, 1971) | 1994.1  (1993.7, 1994.5) |
| - | Central Asia | 0.979 | -2096.8  (6750.6) | 3.3  (3.4) | 34.9  (5.3) | -37.3  (4.2) | 1998.2  (1998.1, 1998.4) | 2001.4  (2001.2, 2001.6) |
| - | East Asia | 0.997 | 2090.9  (1707.5) | 1.8  (0.9) | 763.2  (39.0) | -756.6  (39.0) | 1984  (1984, 1984) | 1985.1  (1985.1, 1985.1) |
| - | South-east Asia | 0.980 | -5659.6  (1213.3) | 5.7  (0.6) | 18.7  (3.7) | -24.6  (3.9) | 1984.4  (1984.2, 1984.6) | 1991.2  (1991, 1991.4) |
| - | South Asia | 0.991 | -6102.7  (732.8) | 6.1  (0.4) | 56.1  (5.7) | -62.2  (5.9) | 1988.8  (1988.8, 1988.9) | 1990.1  (1990.1, 1990.2) |
| - | Western Asia | 0.976 | 2115.3  (5259.9) | 1.7  (2.7) | 20.2  (2.9) | -15.6  (5.0) | 1975.5  (1975.5, 1975.5) | 2004.8  (2004.8, 2004.8) |
| - | Eastern Europe | 0.879 | 5207.2  (11343.1) | -0.4  (5.8) | 16.0  (5.9) | -39.0  (24.6) | 1973.4  (1973.4, 1973.4) | 2009.7  (2009.7, 2009.7) |
| - | Northern Europe | 0.970 | -3193.0  (2903.2) | 3.1  (1.5) | 107.3  (18.9) | -97.1  (18.9) | 1987.3  (1986.5, 1988) | 1992  (1991.3, 1992.7) |
| - | Southern Europe | 0.967 | 26492.2  (3009.8) | -10.6  (1.5) | 79.4  (7.1) | -68.2  (6.9) | 1979.7  (1979.6, 1979.8) | 1986.4  (1986.3, 1986.5) |
| - | Western Europe | 0.955 | -9611.2  (2914.9) | 7.0  (1.5) | 66.9  (22.5) | -69.0  (22.4) | 1983.9  (1983.6, 1984.1) | 1988.3  (1988.1, 1988.6) |
| - | Aust. & New Zeal. | 0.970 | -54109.5  (2307.9) | 29.7  (1.2) | -33.7  (6.2) | 13.5  (7.1) | 1971.4  (1971.1, 1971.8) | 1985.4  (1985.1, 1985.8) |
| - | Melanesia | 0.978 | -10806.3  (3691.7) | 7.1  (1.9) | 9.2  (2.0) | -16.3  (2.7) | 1977.2  (1976.8, 1977.6) | 1985.6  (1985.2, 1986) |
| - | Micronesia | 0.462 | -14372.1  (3735.7) | 8.2  (1.9) | -61.1  (25.9) | 70.5  (26.4) | 1987.6  (1987.2, 1988) | 1991.3  (1990.9, 1991.7) |
| - | Polynesia | 0.928 | -14303.3  (1404.9) | 9  (0.7) | -9.0  (1.3) | 1.0  (1.2) | 1976.7  (1976.7, 1976.7) | 1988.9  (1988.9, 1988.9) |
| Simpson diversity | World | 0.931 | 1.5  (0.1) | -0.0003  (0.00001) | 0.0017  (0.0003) | -0.0009  (0.0003) | 1981  (1980.7, 1981.3) | 1986  (1985.7, 1986.4) |
| - | Eastern Africa | 0.708 | 2.0  (0.1) | -0.0005  (0.0001) | 0.0013  (0.0003) | -0.0027  (0.0009) | 1988.6  (1988.1, 1989.1) | 2000.9  (2000.4, 2001.4) |
| - | Middle Africa | 0.693 | 0.0  (0.5) | 0.0005  (0.0002) | -0.0011  (0.0003) | 0.0013  (0.0002) | 1971.5  (1970.8, 1972.3) | 1981.1  (1980.3, 1981.8) |
| - | Northern Africa | 0.743 | -3.5  (1.1) | 0.0022  (0.0006) | -0.0018  (0.0006) | -0.0082  (0.0034) | 1977.1  (1976.7, 1977.5) | 2002  (2001.6, 2002.4) |
| - | Southern Africa | 0.446 | -5.6  (7) | 0.0032  (0.0036) | -0.0027  (0.0036) | 0.0018  (0.0016) | 1973.1  (1972.1, 1974.1) | 1992.2  (1991.3, 1993.2) |
| - | West Africa | 0.780 | -1.3  (0.3) | 0.0011  (0.0002) | -0.0027  (0.0007) | 0.003  (0.0007) | 1983.1  (1982.9, 1983.2) | 1988  (1987.9, 1988.1) |
| - | Caribbean | 0.917 | -0.9  (0.9) | 0.0009  (0.0005) | 0.0012  (0.0008) | 0.0062  (0.0019) | 1979  (1978.4, 1979.6) | 1997.6  (1996.9, 1998.2) |
| - | Central America | 0.804 | -7.6  (0.8) | 0.0042  (0.0004) | -0.0063  (0.001) | 0.0043  (0.0015) | 1985.1  (1984.4, 1985.8) | 1993  (1992.2, 1993.7) |
| - | North America | 0.648 | -1.1  (1.2) | 0.001  (0.0006) | -0.0083  (0.0039) | 0.0075  (0.0038) | 1971.6  (1971, 1972.2) | 1980.4  (1979.8, 1981) |
| - | South America | 0.968 | -0.1  (0.2) | 0.0005  (0.0001) | -0.0015  (0.0002) | -0.0076  (0.0006) | 1983.1  (1983, 1983.3) | 1999.4  (1999.2, 1999.6) |
| - | Central Asia | 0.953 | 46  (5.5) | -0.0227  (0.0027) | 0.0182  (0.0029) | 0.0235  (0.0027) | 1998.7  (1998.6, 1998.9) | 2009.4  (2009.2, 2009.5) |
| - | East Asia | 0.965 | 5.3  (0.4) | -0.0022  (0.0002) | 0.0035  (0.0003) | 0.0009  (0.0002) | 1976.1  (1976, 1976.2) | 1996.8  (1996.8, 1996.9) |
| - | South-east Asia | 0.986 | -6.1  (0.2) | 0.0034  (0.0001) | -0.003  (0.0003) | 0.0031  (0.0007) | 1987.4  (1986.8, 1988) | 1999  (1998.4, 1999.5) |
| - | South Asia | 0.372 | 3.0  (0.9) | -0.0011  (0.0005) | 0.001  (0.0005) | 0.0012  (0.0008) | 1977.5  (1976.8, 1978.1) | 1994.7  (1994.1, 1995.4) |
| - | Western Asia | 0.863 | 2.8  (0.6) | -0.001  (0.0003) | 0.0044  (0.0007) | -0.0036  (0.0007) | 1975.1  (1974.9, 1975.3) | 1987.9  (1987.7, 1988.1) |
| - | Eastern Europe | 0.775 | 0.9  (0.8) | -0.00004  (0.0004) | 0.0031  (0.0006) | -0.0056  (0.0007) | 1967.5  (1967, 1967.9) | 1992.8  (1992.3, 1993.2) |
| - | Northern Europe | 0.916 | 9.9  (1.0) | -0.0047  (0.0005) | 0.0199  (0.0022) | -0.0145  (0.0022) | 1978.8  (1978.7, 1979) | 1989.8  (1989.6, 1989.9) |
| - | Southern Europe | 0.977 | 1.8  (1.2) | -0.0005  (0.0006) | 0.0026  (0.0006) | -0.002  (0.0002) | 1966.8  (1966.4, 1967.3) | 1988  (1987.5, 1988.5) |
| - | Western Europe | 0.852 | 3.7  (0.3) | -0.0014  (0.0001) | 0.0077  (0.0014) | -0.0076  (0.0014) | 1982.8  (1982.7, 1982.9) | 1987.8  (1987.6, 1987.9) |
| - | Aust. & New Zeal. | 0.866 | -5.4  (1.9) | 0.003  (0.0009) | 0.0209  (0.0073) | -0.0271  (0.0074) | 1982.4  (1982, 1982.8) | 1991.6  (1991.2, 1992.1) |
| - | Melanesia | 0.982 | -10.0  (0.5) | 0.0054  (0.0003) | -0.0067  (0.0005) | 0.0056  (0.0006) | 1982.2  (1982, 1982.3) | 1990.5  (1990.4, 1990.6) |
| - | Micronesia | 0.632 | 0.5  (0.8) | -0.0002  (0.0004) | -0.0032  (0.0015) | 0.0092  (0.0017) | 1980  (1979.5, 1980.4) | 1991.9  (1991.5, 1992.4) |
| - | Polynesia | 0.643 | 25.2  (10.2) | -0.0125  (0.0052) | 0.0124  (0.0052) | 0.0047  (0.0013) | 1968.7  (1968.1, 1969.3) | 1987.3  (1986.7, 1987.9) |
| QE_phy_ | Word | 0.991 | 111.3  (44.3) | -0.014  (0.01) | 0.5  (0.001) | -0.2  (0.3) | 1968.2  (1967.8, 1968.6) | 1981.6  (1981.2, 1982) |
| - | Eastern Africa | 0.69 | 217.9  (314.3) | -0.1  (0.2) | 0.5  (0.2) | -0.3  (0.2) | 1991.8  (1990.8, 1992.7) | 1995.9  (1995, 1996.8) |
| - | Middle Africa | 0.887 | -359.9  (112) | 0.2  (0.1) | -0.3  (0.1) | -0.2  (0.1) | 1971  (1970.8, 1971.3) | 1978.4  (1978.2, 1978.6) |
| - | Northern Africa | 0.253 | -919.5  (424.4) | 0.5  (0.2) | -0.9  (0.3) | 0.5  (0.2) | 1976.9  (1976.1, 1977.7) | 1992  (1991.2, 1992.8) |
| - | Southern Africa | 0.867 | -59.4  (124.2) | 0.1  (0.1) | 1.0  (0.1) | -2.9  (3.5) | 1985.7  (1985.1, 1986.3) | 1998.7  (1998.1, 1999.3) |
| - | West Africa | 0.831 | -422.5  (55.3) | 0.3  (0.001) | -2.1  (0.4) | 2.3  (0.4) | 1982.2  (1982.1, 1982.4) | 1987.3  (1987.1, 1987.5) |
| - | Caribbean | 0.932 | 442.3  (319.0) | -0.2  (0.2) | 0.5  (0.2) | 2.4  (0.4) | 1971  (1970.6, 1971.4) | 2000.7  (2000.3, 2001.1) |
| - | Central America | 0.499 | -367.7  (140.8) | 0.2  (0.1) | -0.3  (0.1) | 0.4  (0.2) | 1978.8  (1978.2, 1979.4) | 1984.7  (1984.1, 1985.3) |
| - | North America | 0.869 | -1507.5  (219.6) | 0.8  (0.1) | -3.4  (3.4) | 3.2  (3.4) | 1979  (1978.4, 1979.5) | 1985.8  (1985.3, 1986.3) |
| - | South America | 0.838 | -171.5  (126.8) | 0.1  (0.1) | 0.3  (0.1) | -0.6  (0.1) | 1965.3  (1964.5, 1966.2) | 1995.2  (1994.3, 1996.1) |
| - | Central Asia | 0.541 | -1055.9  (444.5) | 0.6  (0.2) | -1.2  (0.5) | 2.2  (0.7) | 1999  (1998.7, 1999.3) | 2008.9  (2008.6, 2009.2) |
| - | East Asia | 0.977 | 1014.2  (137.7) | -0.5  (0.1) | 1.2  (0.1) | 0.2  (0.3) | 1974.9  (1974.8, 1975) | 1997.6  (1997.5, 1997.7) |
| - | South-east Asia | 0.965 | -634.1  (28.5) | 0.4  (0.001) | -0.8  (1.1) | 1.2  (1.1) | 1992.7  (1991.9, 1993.6) | 1998.1  (1997.3, 1999) |
| - | South Asia | 0.922 | 311.1  (46.2) | -0.1  (0.001) | 0.6  (0.1) | -0.7  (0.1) | 1976.5  (1976, 1976.9) | 1994.4  (1993.9, 1994.8) |
| - | Western Asia | 0.936 | -735.2  (54.2) | 0.4  (0.001) | -0.2  (0.1) | -2.0  (0.5) | 1980.5  (1979.7, 1981.3) | 2000  (1999.2, 2000.8) |
| - | Eastern Europe | 0.728 | 666.7  (161.1) | -0.3  (0.1) | 1.4  (0.5) | -0.8  (0.5) | 1978.4  (1977.8, 1979) | 1989.8  (1989.2, 1990.4) |
| - | Northern Europe | 0.957 | 678.7  (112.2) | -0.3  (0.1) | 2.5  (0.2) | -2.2  (0.2) | 1978.5  (1978.3, 1978.6) | 1988.3  (1988.2, 1988.5) |
| - | Southern Europe | 0.979 | 747.7  (170.8) | -0.3  (0.1) | 0.9  (0.1) | -0.8  (0.1) | 1970.2  (1970.1, 1970.2) | 1993.1  (1993, 1993.1) |
| - | Western Europe | 0.963 | 1004.0  (76.0) | -0.5  (0.001) | 1.9  (0.2) | -1.7  (0.2) | 1980.2  (1980.1, 1980.3) | 1989.8  (1989.7, 1989.9) |
| - | Aust. & New Zeal. | 0.951 | -1272.4  (315.6) | 0.7  (0.2) | 1.2  (0.3) | -4.3  (0.6) | 1981.2  (1981.1, 1981.4) | 1997.5  (1997.4, 1997.7) |
| - | Melanesia | 0.970 | -1146.5  (53.0) | 0.6  (0.001) | -1.2  (0.1) | 1.0  (0.1) | 1980.1  (1979.9, 1980.3) | 1993.6  (1993.4, 1993.8) |
| - | Micronesia | 0.676 | -500.8  (141.2) | 0.3  (0.1) | -0.8  (0.1) | 1.5  (0.2) | 1980.1  (1979.7, 1980.5) | 1991.9  (1991.5, 1992.3) |
| - | Polynesia | 0.620 | 6075.6  (6603.5) | -3.1  (3.4) | 3.1  (3.4) | 0.4  (0.2) | 1968.7  (1968, 1969.4) | 1987.9  (1987.2, 1988.6) |
| Ag. area | Word | 0.997 | -14123594.4  (653567.1) | 9482.7  (331.3) | 11555.2  (704.4) | -23395.3  (1401.9) | 1982.8  (1982.8, 1982.9) | 1995.3  (1995.2, 1995.3) |
| - | Eastern Africa | 0.979 | -808538.4  (33931.5) | 556.9  (17.2) | -18198.7  (1241.6) | 19379.9  (1243.8) | 1991.9  (1991.8, 1992) | 1993.2  (1993.1, 1993.4) |
| - | Middle Africa | 0.997 | -110349  (4210.9) | 137.1  (2.1) | -82.8  (15.2) | -56.6  (17.7) | 1985.5  (1985, 1985.9) | 2002.2  (2001.8, 2002.7) |
| - | Northern Africa | 0.989 | -594943.4  (63211.6) | 408  (32.1) | 1514.4  (171.5) | -1192.9  (217.2) | 1985  (1984.8, 1985.2) | 1994.9  (1994.7, 1995.1) |
| - | Southern Africa | 0.915 | 1210923.5  (102755.2) | -531.4  (52.3) | 773.0  (55.8) | -385.5  (92.1) | 1978.7  (1978.1, 1979.3) | 1997.6  (1997, 1998.1) |
| - | West Africa | 0.992 | -2145489.1  (329472) | 1209.5  (167.7) | -2349.2  (235.6) | 3423.2  (171.3) | 1969.1  (1968.6, 1969.6) | 1980.5  (1980, 1981) |
| - | Caribbean | 0.983 | -339237.1  (22217.4) | 177.6  (11.3) | -104.5  (12.5) | -108.4  (9.1) | 1973.1  (1972.8, 1973.5) | 1989  (1988.6, 1989.3) |
| - | Central America | 0.995 | -127606.6  (41302.8) | 121.8  (21.0) | 803.3  (44.9) | -818.7  (51) | 1978.7  (1978.5, 1978.8) | 1991.5  (1991.4, 1991.7) |
| - | North America | 0.985 | 3017623.3  (163427.4) | -1276.5  (83.1) | 1804.9  (237.9) | -1561.8  (227.9) | 1979.4  (1979, 1979.8) | 1984.6  (1984.1, 1985) |
| - | South America | 0.998 | -9057595.4  (291867.7) | 4844.3  (148.4) | -2021.9  (171.5) | -769.9  (304.1) | 1975.3  (1975.2, 1975.5) | 1997.7  (1997.5, 1997.9) |
| - | Central Asia | 0.942 | 8743180  (2050598.1) | -4234.5  (1028.2) | 3653.1  (1029.5) | 1706.5  (224.3) | 1995.9  (1995.6, 1996.2) | 2007.9  (2007.6, 2008.2) |
| - | East Asia | 0.991 | -7125318.6  (749419.8) | 3885.0  (380.8) | 1725.1  (434.6) | -9593.7  (842.9) | 1979.5  (1979.4, 1979.6) | 1991.8  (1991.7, 1992) |
| - | South-east Asia | 0.987 | -740564.7  (169473.2) | 420.2  (86.2) | 377.5  (91.3) | 762.9  (433.8) | 1972  (1971.5, 1972.4) | 1999.5  (1999.1, 2000) |
| - | South Asia | 0.962 | -3083449.2  (2321803.0) | 1737  (1183.8) | -1472.2  (1183.9) | -9015.9  (483.5) | 1965.7  (1964.9, 1966.6) | 2002.9  (2002, 2003.7) |
| - | Western Asia | 0.973 | -189575.3  (342156.4) | 185.3  (173.5) | 10013.9  (947.3) | -9782.8  (1235.0) | 1986.4  (1986.2, 1986.6) | 1994.3  (1994.2, 1994.5) |
| - | Eastern Europe | 0.999 | -294776.8  (50099.2) | 455.1  (25.3) | -280706.1  (1368.7) | 279532.4  (1374.6) | 1991  (1990.9, 1991.1) | 1992  (1991.9, 1992.1) |
| - | Northern Europe | 0.791 | 324705.9  (49925.5) | -145.8  (25.4) | 1730.1  (223.3) | -1895.9  (231.3) | 1988.4  (1988, 1988.8) | 1993.3  (1992.9, 1993.7) |
| - | Southern Europe | 0.991 | 847223.4  (69003.0) | -389.3  (35.1) | 213.9  (55.2) | -355.5  (46.8) | 1975.8  (1975.7, 1975.9) | 1988.1  (1987.9, 1988.2) |
| - | Western Europe | 0.995 | 671550.3  (47290.3) | -309.9  (24.0) | 90.3  (25.1) | 84.3  (18.7) | 1970.9  (1970.4, 1971.5) | 1993.3  (1992.8, 1993.9) |
| - | Aust. & New Zeal. | 0.956 | -3711662.4  (495986.7) | 2136.5  (252.4) | -3266.4  (271.7) | -2795.2  (524.6) | 1973.3  (1973.2, 1973.3) | 1998.3  (1998.3, 1998.4) |
| - | Melanesia | 0.998 | -9147.8  (10258.5) | 5.2  (5.2) | 16.8  (5.2) | -22.3  (7.0) | 1964.2  (1963.4, 1965.1) | 1997.5  (1996.6, 1998.3) |
| - | Micronesia | 0.941 | 1526.0  (117.9) | -0.7  (0.1) | 0.9  (0.1) | -0.6  (0.1) | 1978.2  (1977.8, 1978.6) | 1983.6  (1983.2, 1984) |
| - | Polynesia | 0.975 | -2651.0  (136.8) | 1.4  (0.1) | -7.4  (0.5) | 5.3  (0.5) | 1985.8  (1985.4, 1986.2) | 1991.1  (1990.8, 1991.5) |

**Table D. Indicators of temporal change in crop diversity and agriculture area across 22 regions.** Acronyms for diversity indices are as follows: QE_phy_=phylogenetic Rao’s quadratic entropy, and Ag. Area=agricultural area. Region names follow those of the Food and Agricultural Organization of the United Nations. Indicators are based on piecewise model fits such that the “Timing of 1^st^ breakpoint” represents the timing of the first major breakpoint in crop diversity (or agricultural area) change (i.e. ψ1 in Equation 3 in main text; measured as a year); “Rate of change” represents the change in the slope of the relationship between crop *D* (or agricultural area) and year that occurs at/ beyond the time of the first major breakpoint (i.e. *b* + *c* from Equation 3 in main text; units the same as the metric of *D* or in ha year^-1^ for agricultural area); and “Duration of major change” represents the duration of the period of major change in crop *D* (or agricultural area) through time (i.e. calculated as ψ1-ψ2 from Equation 3 in main text; years). Piecewise model fits from which indicators were derived are presented in S1 File Table B.

| **Metric** | **Region** | **Timing of 1^st^ breakpoint** | **Rate of change** | **Duration of major change** |
| --- | --- | --- | --- | --- |
| Species richness | World | 1967.9 | 0.2 | 24.0 |
| - | Eastern Africa | 1982.8 | 1.00 | 12.1 |
| - | Middle Africa | 1982.7 | 2.08 | 8.7 |
| - | Northern Africa | 1983.6 | 1.69 | 6.3 |
| - | Southern Africa | 1975.6 | 0.25 | 19.8 |
| - | West Africa | 1984.8 | 1.86 | 7.5 |
| - | Caribbean | 1984.0 | 10.44 | 1.8 |
| - | Central America | 1977.2 | 2.32 | 9.6 |
| - | North America | 1975.9 | 0.50 | 16.9 |
| - | South America | 1981.4 | 1.04 | 10.9 |
| - | Central Asia | 1999.1 | 2.00 | 2.1 |
| - | East Asia | 1983.5 | 8.07 | 2.1 |
| - | South-east Asia | 1985.3 | 1.46 | 5.9 |
| - | South Asia | 1987.0 | 4.02 | 4.1 |
| - | Western Asia | 1975.7 | 0.79 | 5.3 |
| - | Eastern Europe | 1981.9 | 1.18 | 7.8 |
| - | Northern Europe | 1989.8 | 4.49 | 2.4 |
| - | Southern Europe | 1979.5 | 1.07 | 8.7 |
| - | Western Europe | 1983.8 | 1.50 | 4.2 |
| - | Aust. & New Zeal. | 1976.7 | -0.36 | 8.9 |
| - | Melanesia | 1976.3 | 0.35 | 18.7 |
| - | Micronesia | 1986.9 | -0.67 | 4.7 |
| - | Polynesia | 1978.4 | -0.00002 | 15.5 |
| Phylogenetic diversity | Word | 1980.6 | 29.46 | 4.9 |
| - | Eastern Africa | 1988.2 | 74.02 | 4.4 |
| - | Middle Africa | 1982.6 | 82.05 | 8.4 |
| - | Northern Africa | 1983.3 | 74.89 | 4.9 |
| - | Southern Africa | 1978.7 | 11.36 | 25.1 |
| - | West Africa | 1986.0 | 64.05 | 6.9 |
| - | Caribbean | 1984.0 | 467.26 | 1.1 |
| - | Central America | 1977.5 | 93.91 | 9.1 |
| - | North America | 1983.9 | 19.65 | 20.4 |
| - | South America | 1970.6 | 34.89 | 23.4 |
| - | Central Asia | 1998.2 | 38.23 | 3.2 |
| - | East Asia | 1984.0 | 765.08 | 1.1 |
| - | South-east Asia | 1984.4 | 24.40 | 6.8 |
| - | South Asia | 1988.8 | 62.20 | 1.3 |
| - | Western Asia | 1975.5 | 21.95 | 29.3 |
| - | Eastern Europe | 1973.4 | 15.62 | 36.3 |
| - | Northern Europe | 1987.3 | 110.36 | 4.8 |
| - | Southern Europe | 1979.7 | 68.74 | 6.7 |
| - | Western Europe | 1983.9 | 73.81 | 4.5 |
| - | Aust. & New Zeal. | 1971.4 | -4.05 | 14.0 |
| - | Melanesia | 1977.2 | 16.32 | 8.3 |
| - | Micronesia | 1987.6 | -52.87 | 3.7 |
| - | Polynesia | 1976.7 | -0.01 | 12.2 |
| Simpson diversity | Word | 1981.0 | 0.001 | 5.0 |
| - | Eastern Africa | 1988.6 | 0.001 | 12.3 |
| - | Middle Africa | 1971.5 | -0.001 | 9.5 |
| - | Northern Africa | 1977.1 | 0.0004 | 24.9 |
| - | Southern Africa | 1973.1 | 0.0005 | 19.1 |
| - | West Africa | 1983.1 | -0.002 | 4.9 |
| - | Caribbean | 1979.0 | 0.002 | 18.6 |
| - | Central America | 1985.1 | -0.002 | 7.9 |
| - | North America | 1971.6 | -0.007 | 8.8 |
| - | South America | 1983.1 | -0.001 | 16.2 |
| - | Central Asia | 1998.7 | -0.005 | 10.7 |
| - | East Asia | 1976.1 | 0.001 | 20.7 |
| - | South-east Asia | 1987.4 | 0.0004 | 11.6 |
| - | South Asia | 1977.5 | -0.00002 | 17.2 |
| - | Western Asia | 1975.1 | 0.003 | 12.8 |
| - | Eastern Europe | 1967.5 | 0.003 | 25.3 |
| - | Northern Europe | 1978.8 | 0.015 | 10.9 |
| - | Southern Europe | 1966.8 | 0.002 | 21.2 |
| - | Western Europe | 1982.8 | 0.006 | 5.0 |
| - | Aust. & New Zeal. | 1982.4 | 0.024 | 9.3 |
| - | Melanesia | 1982.2 | -0.001 | 8.3 |
| - | Micronesia | 1980.0 | -0.003 | 12.0 |
| - | Polynesia | 1968.7 | -0.0002 | 18.6 |
| QE_phy_ | World | 1968.2 | 0.46 | 13.4 |
| - | Eastern Africa | 1991.8 | 0.41 | 4.1 |
| - | Middle Africa | 1971.0 | -0.10 | 7.4 |
| - | Northern Africa | 1976.9 | -0.40 | 15.1 |
| - | Southern Africa | 1985.7 | 1.05 | 13.0 |
| - | West Africa | 1982.2 | -1.81 | 5.0 |
| - | Caribbean | 1971.0 | 0.29 | 29.7 |
| - | Central America | 1978.8 | -0.07 | 5.9 |
| - | North America | 1979.0 | -2.58 | 6.8 |
| - | South America | 1965.3 | 0.43 | 29.9 |
| - | Central Asia | 1999.0 | -0.66 | 9.8 |
| - | East Asia | 1974.9 | 0.69 | 22.7 |
| - | South-east Asia | 1992.7 | -0.42 | 5.4 |
| - | South Asia | 1976.5 | 0.47 | 17.9 |
| - | Western Asia | 1980.5 | 0.20 | 19.5 |
| - | Eastern Europe | 1978.4 | 1.06 | 11.4 |
| - | Northern Europe | 1978.5 | 2.17 | 9.9 |
| - | Southern Europe | 1970.2 | 0.55 | 22.9 |
| - | Western Europe | 1980.2 | 1.45 | 9.6 |
| - | Aust. & New Zeal. | 1981.2 | 1.88 | 16.3 |
| - | Melanesia | 1980.1 | -0.55 | 13.5 |
| - | Micronesia | 1980.1 | -0.50 | 11.8 |
| - | Polynesia | 1968.7 | 0.05 | 19.3 |
| Ag. area | World | 1982.8 | 21038 | 12.4 |
| - | Eastern Africa | 1991.9 | -17641.8 | 1.4 |
| - | Middle Africa | 1985.5 | 54.3 | 16.7 |
| - | Northern Africa | 1985.0 | 1922.4 | 9.9 |
| - | Southern Africa | 1978.7 | 241.7 | 18.9 |
| - | West Africa | 1969.1 | -1139.7 | 11.4 |
| - | Caribbean | 1973.1 | 73.1 | 15.9 |
| - | Central America | 1978.7 | 925 | 12.9 |
| - | North America | 1979.4 | 528.4 | 5.1 |
| - | South America | 1975.3 | 2822.4 | 22.4 |
| - | Central Asia | 1995.9 | -581.4 | 11.9 |
| - | East Asia | 1979.5 | 5610 | 12.3 |
| - | South-east Asia | 1972.0 | 797.8 | 27.6 |
| - | South Asia | 1965.7 | 264.8 | 37.1 |
| - | Western Asia | 1986.4 | 10199.1 | 7.9 |
| - | Eastern Europe | 1991.0 | -280251 | 1.0 |
| - | Northern Europe | 1988.4 | 1584.3 | 4.9 |
| - | Southern Europe | 1975.8 | -175.4 | 12.3 |
| - | Western Europe | 1970.9 | -219.6 | 22.4 |
| - | Aust. & New Zeal. | 1973.3 | -1129.9 | 25.1 |
| - | Melanesia | 1964.2 | 22 | 33.2 |
| - | Micronesia | 1978.2 | 0.14 | 5.4 |
| - | Polynesia | 1985.8 | -5.96 | 5.3 |

**Table E. Trends in standard effect size (SES) of mean pairwise distances through time in agricultural lands of 22 regions, as compared to global data.** Intercepts and slopes are based on an analysis of covariance model (ANCOVA), where SES values were predicted as a function of region and year, and region-by-year interaction term (where *n*=1481 total). All terms in the ANCOVA model were significant (*p*<0.001 in all three cases, overall model *r*^2^=0.97, d.f.=1165). Significant model parameters (*p*≤0.05) are highlighted in bold. Also shown is the number of years within each region (of 54 total in all cases expect 22 in Central Asia) where crops showed significant phylogenetic clustering or over-dispersion, as compared to a random expectation. Agricultural area in 2014 for each region, based on the FAO of the United Nations, are also presented.

| **Region** | **Intercept** | **Slope parameter** | **Years SES clustered** | **Years SES over-dispersed** | **Agricultural area (1000s ha)** |
| --- | --- | --- | --- | --- | --- |
| World (reference) | **-64.89** | **0.03** | 54 | 0 | 4900105.17 |
| Aust. & New Zeal. | **-78.67** | **0.038** | 46 | 0 | 417386.0 |
| Caribbean | **24.27** | **-0.013** | 1 | 0 | 11375.41 |
| Central America | **92.31** | **-0.048** | 35 | 0 | 124628.9 |
| Central Asia | **-138.11** | **0.068** | 2 | 0 | 292902.1 |
| Eastern Africa | **-18.91** | **0.008** | 54 | 0 | 341221.6 |
| Eastern Asia | **-30.05** | **0.013** | 54 | 0 | 637191.5 |
| Eastern Europe | **2.48** | **-0.003** | 54 | 0 | 314801.4 |
| Melanesia | **20.93** | **-0.01** | 0 | 0 | 2094.2 |
| Micronesia | **-2.71** | **0.001** | 0 | 0 | 93.9 |
| Middle Africa | **26.41** | **-0.013** | 0 | 0 | 166274.7 |
| Northern Africa | **3.42** | **-0.003** | 54 | 0 | 174380.7 |
| North America | **-131.91** | **0.065** | 51 | 0 | 473698.9 |
| Northern Europe | **-24.03** | **0.011** | 54 | 0 | 38335.8 |
| Polynesia | **-4.9** | **0.003** | 0 | 0 | 133.3 |
| South-Eastern Asia | **-6.34** | **0.003** | 0 | 0 | 131126.8 |
| South America | **-149.51** | **0.074** | 37 | 0 | 620449.7 |
| Southern Africa | **-25.13** | **0.011** | 54 | 0 | 165150.3 |
| Southern Asia | -64.0973 | 0.03 | 54 | 0 | 316208.7 |
| Southern Europe | **15.1** | **-0.009** | 1 | 0 | 62108.5 |
| Western Africa | **8.73** | **-0.006** | 21 | 0 | 284812.0 |
| Western Asia | **-37.73** | **0.018** | 9 | 0 | 272695.6 |
| Western Europe | -52.77 | 0.025 | 54 | 0 | 53035.1 |


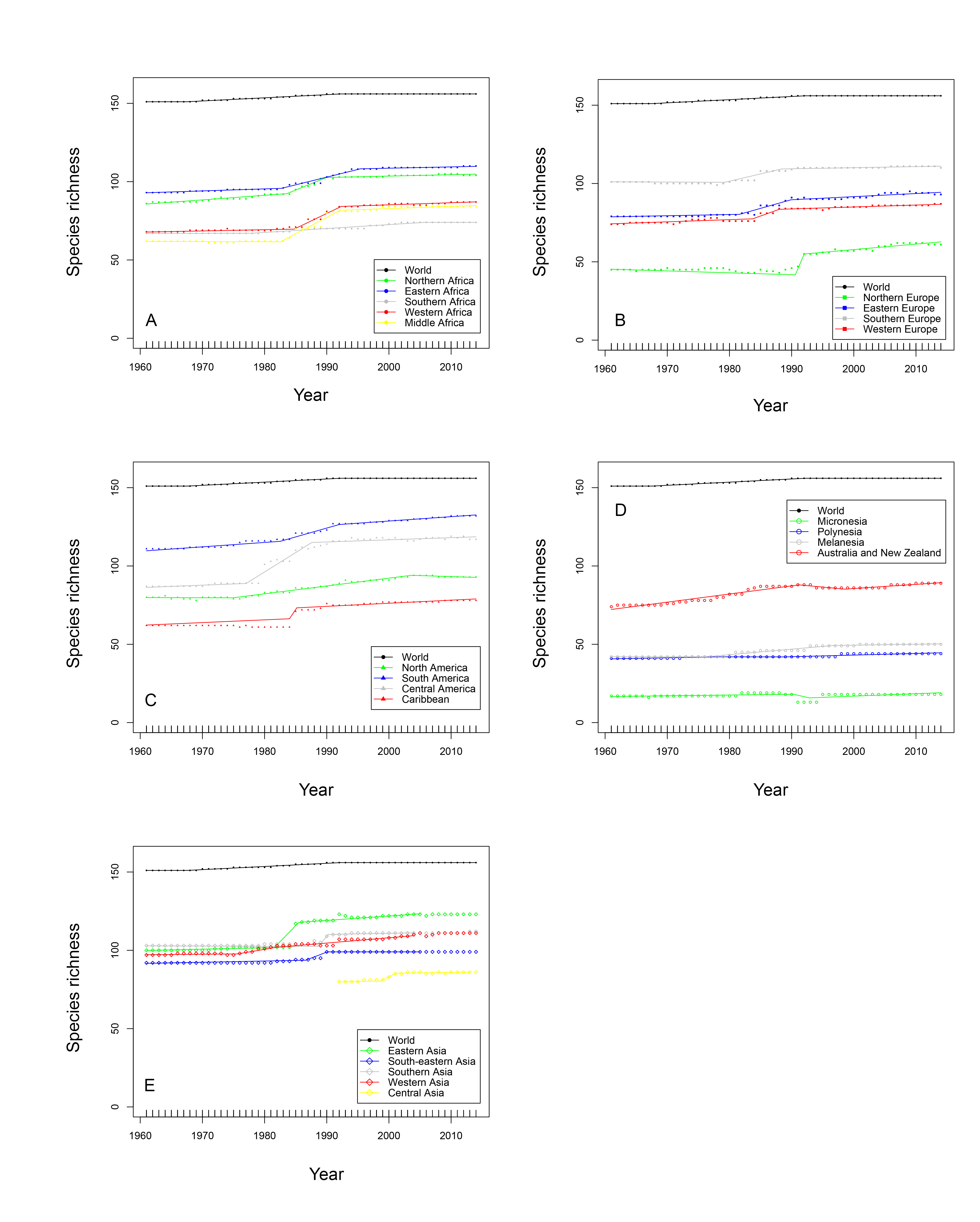


**Fig A. Changes in crop species richness through time across 22 regions.** Trend lines correspond to piecewise linear models fit for each region individually (following Equations 2 and 3 in main text; also shown in black are global trends based on a global FAO dataset analyzed independently). Detailed parameters for each model are presented in S1 File Table C.

**
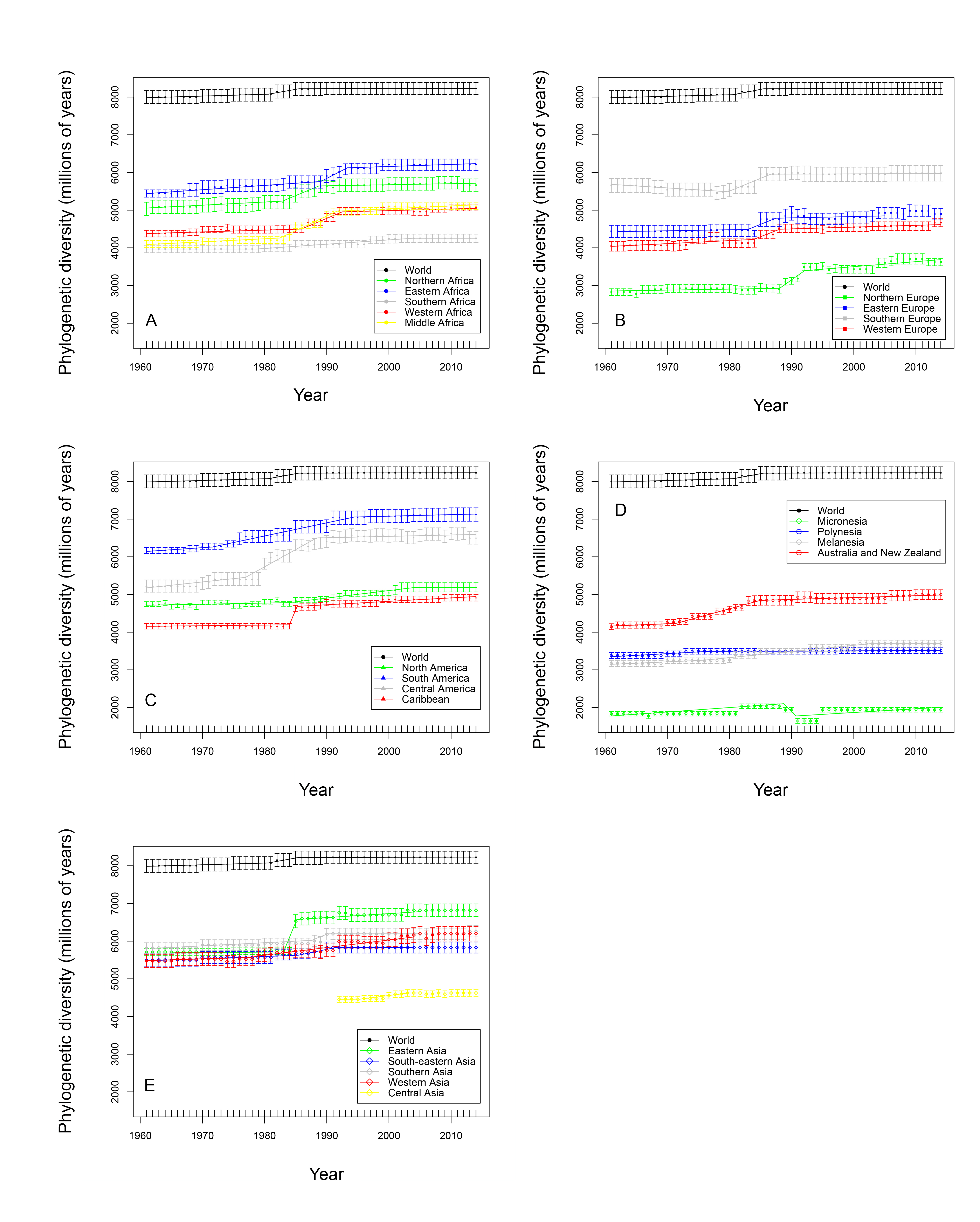
**

**Fig B. Changes in crop species phylogenetic diversity (PD) through time across 22 regions.** Data points correspond to the median values of PD generated through a randomization procedure (see Methods in main text), and error bars correspond to the median absolute deviation surrounding median values. Trend lines correspond to piecewise linear models fit for each region individually (following Equations 2 and 3 in main text; also shown in black are global trends based on a global FAO dataset analyzed independently). Detailed parameters for each model are presented in S1 File Table C.

**
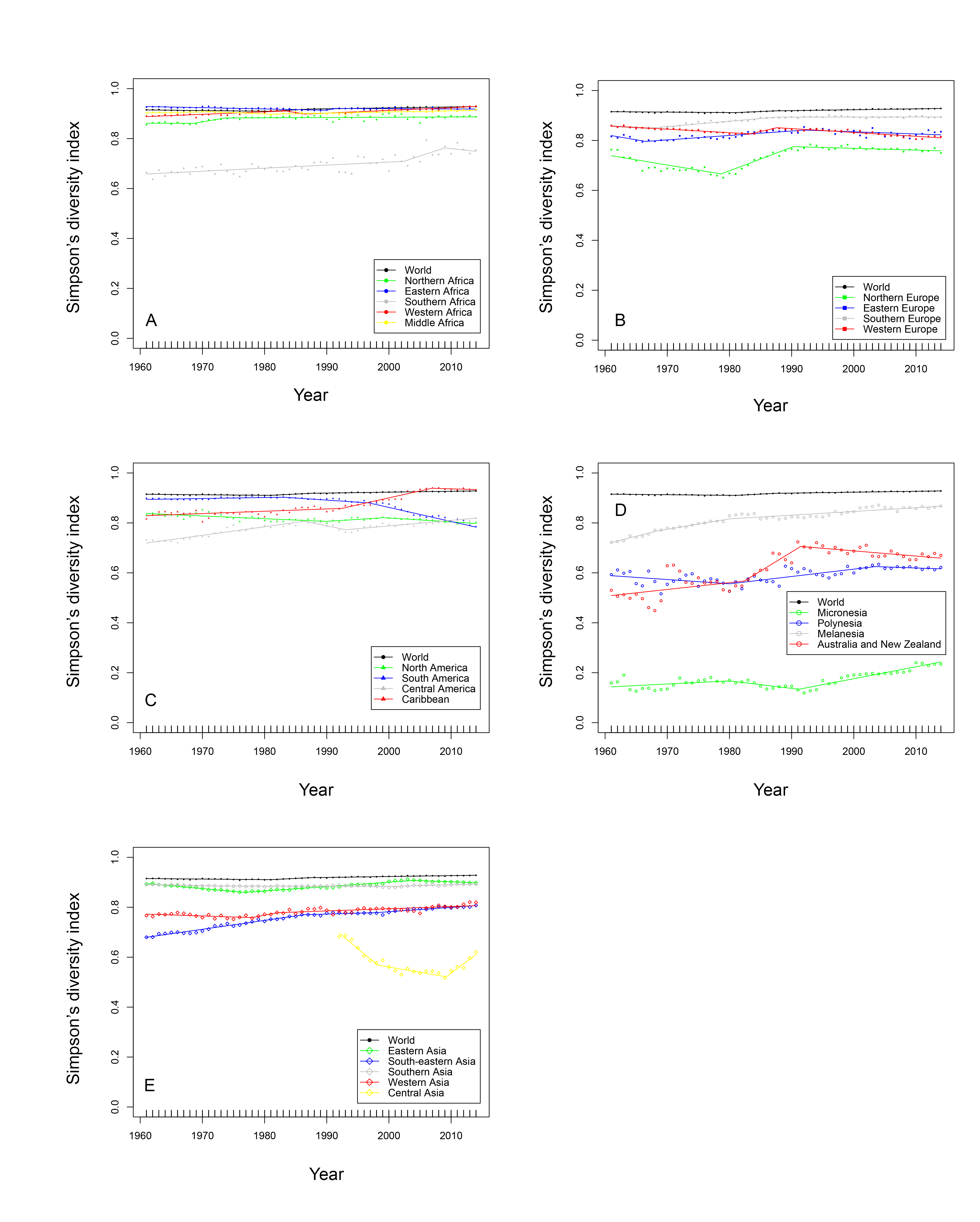
**

**Fig C. Changes in Simpson’s diversity of crops through time across 22 regions.** Trend lines correspond to piecewise linear models fit for each region individually (following Equations 2 and 3). Detailed parameters for each model are presented in S1 File Table C.

**
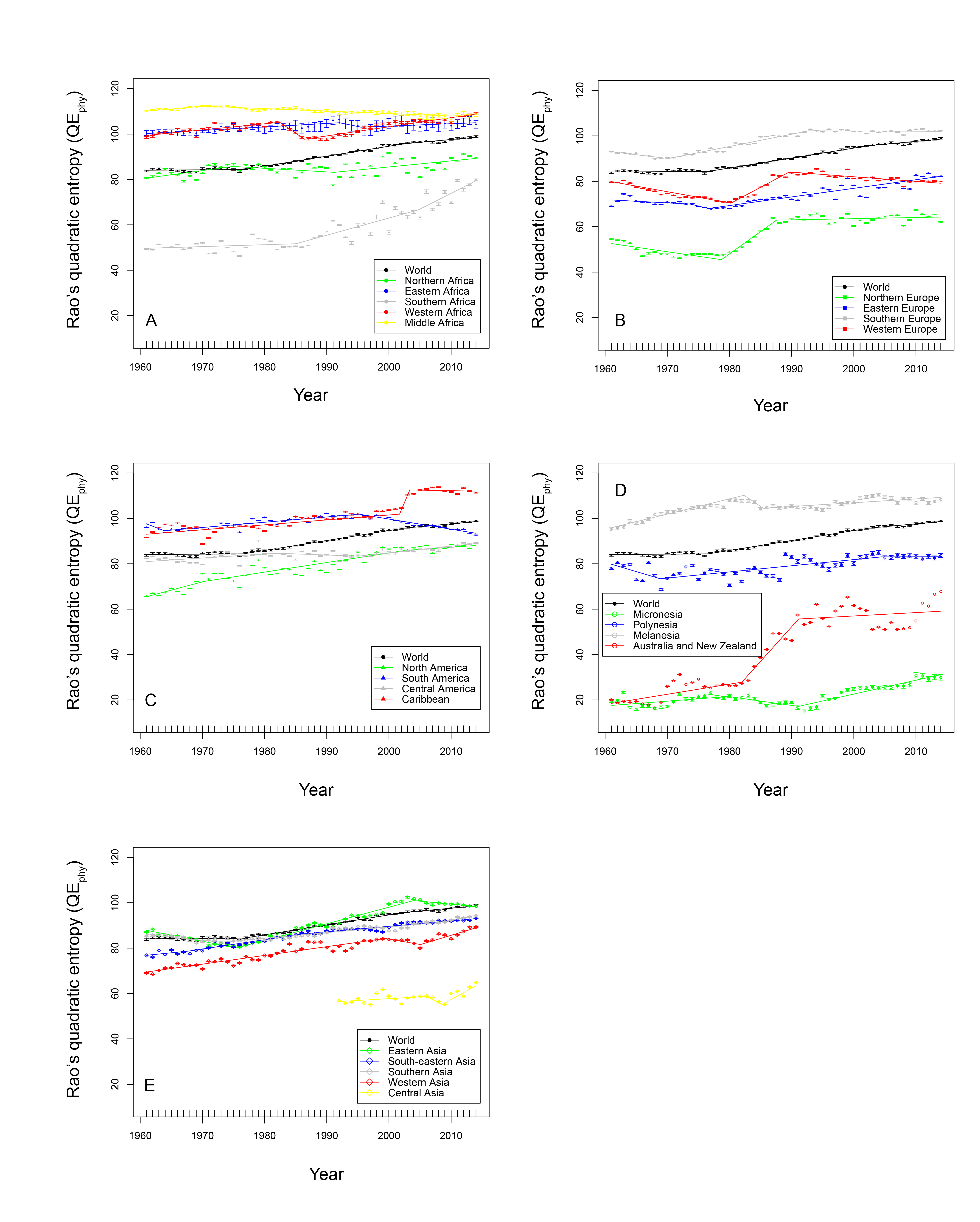
**

**Fig D. Changes in Rao’s quadratic entropy (QE_phy_) in crop species through time across 22 regions.** Data points correspond to the median values of these diversity indices generated through a randomization procedure (see Methods in main text), and error bars correspond to the median absolute deviation surrounding median values. Trend lines correspond to piecewise linear models fit for each region individually (following Equations 2 and 3; also shown in black are global trends based on a global FAO dataset analyzed independently). Detailed parameters for each model are presented in S1 File Table C.

**
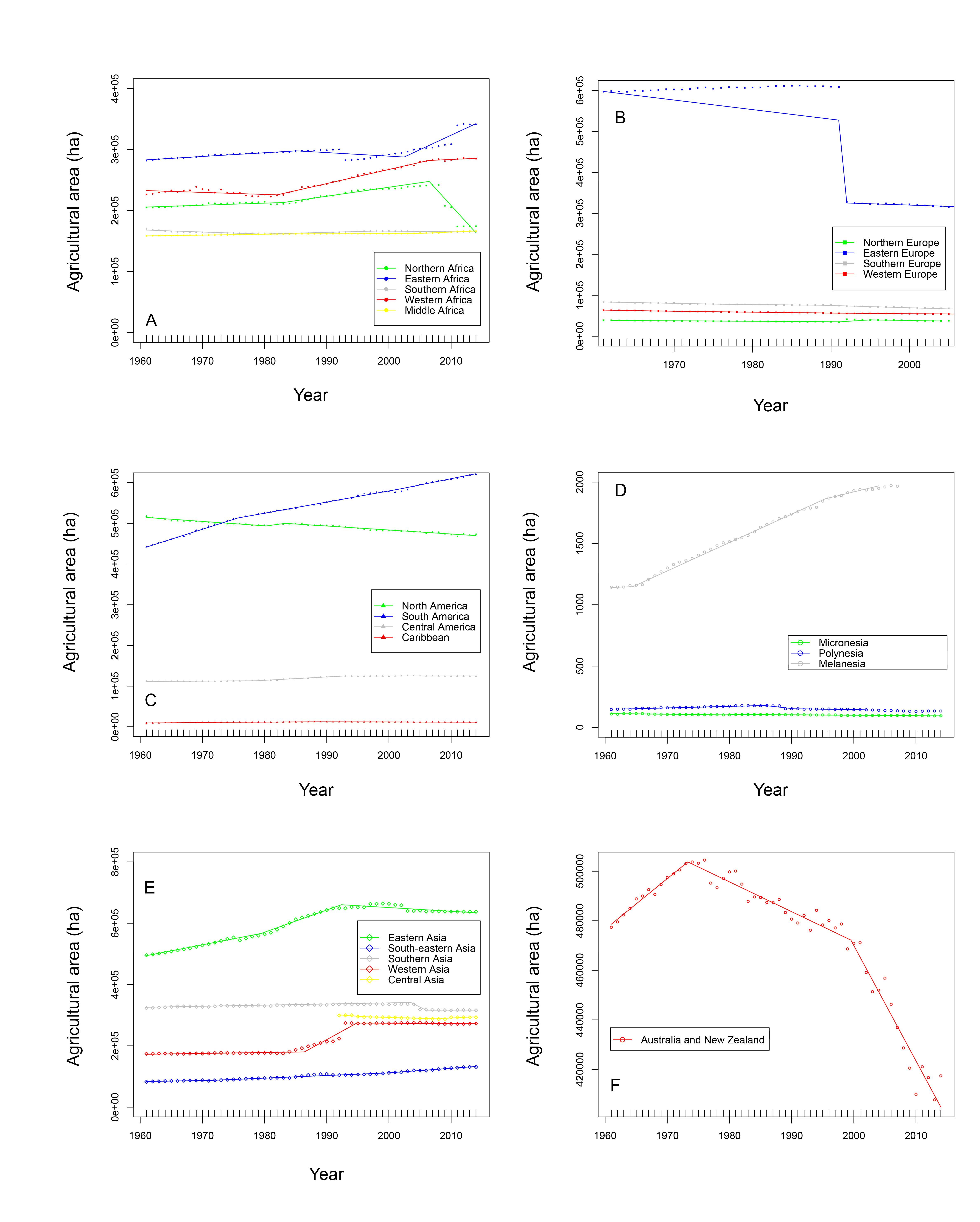
**

**Fig E. Changes in agricultural area through time across 22 regions.** Trend lines correspond to piecewise linear models fit for each region individually (following Equations 2 and 3; also shown in black are global trends based on a global FAO dataset analyzed independently). Detailed parameters for each model are presented in S1 File Table C.

**
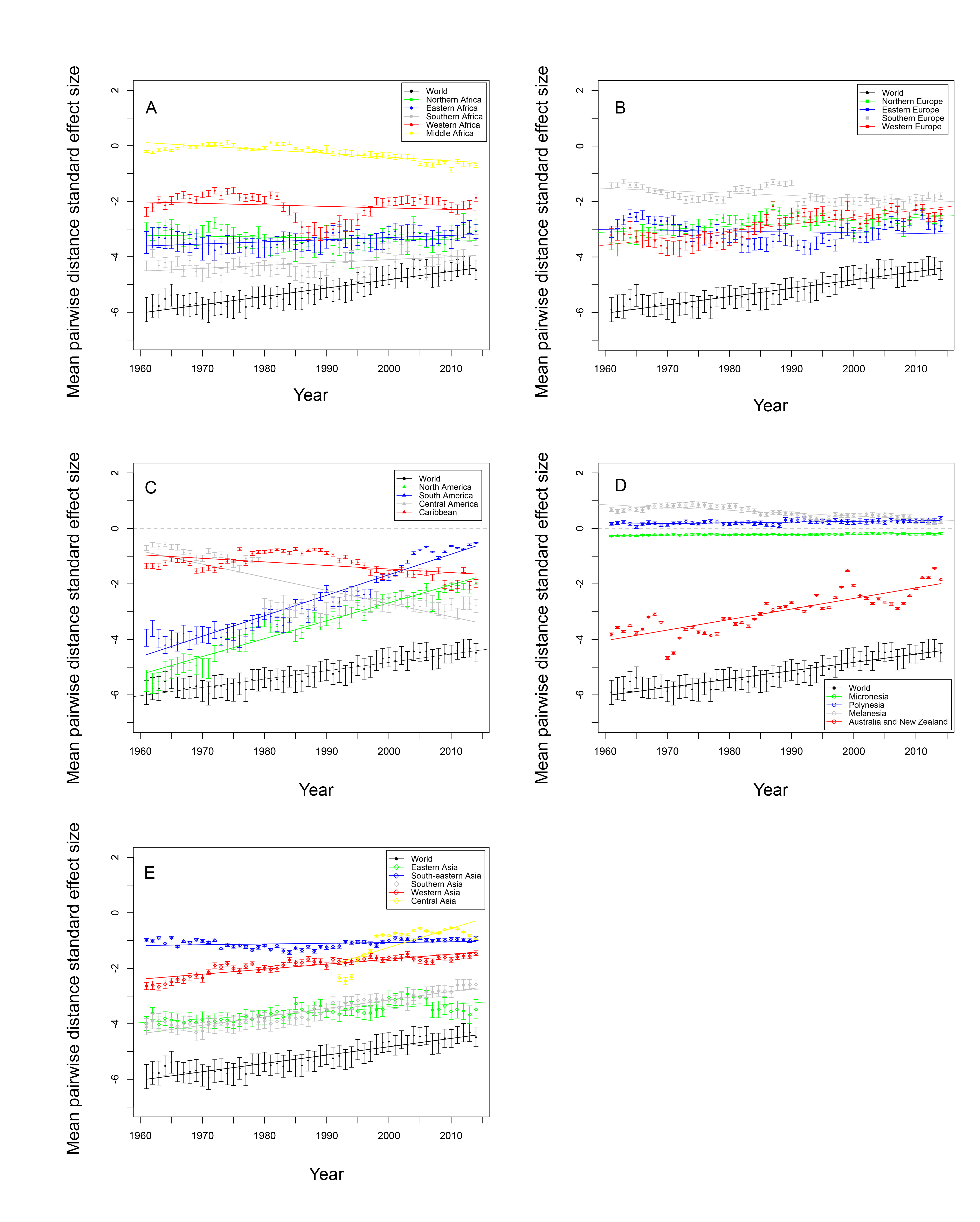
**

**Fig F. Changes in mean pairwise distances of crops through time across 22 regions.** Trend lines correspond to linear models fits describing change in mean pairwise distances through time (following Equations 2; also shown in black are global trends based on a global FAO dataset analyzed independently). Dashed gray trends lines where mean pairwise distance=0 correspond to the difference between i) crop species composition that is more phylogenetically over-dispersed than expected by chance alone (values above the dashed line), vs. ii) crop species composition that is more phylogenetically clustered than expected by chance alone (values below the dashed lines); summaries of these interpretations are presented in Detailed parameters for each model are presented in S1 File Table C.


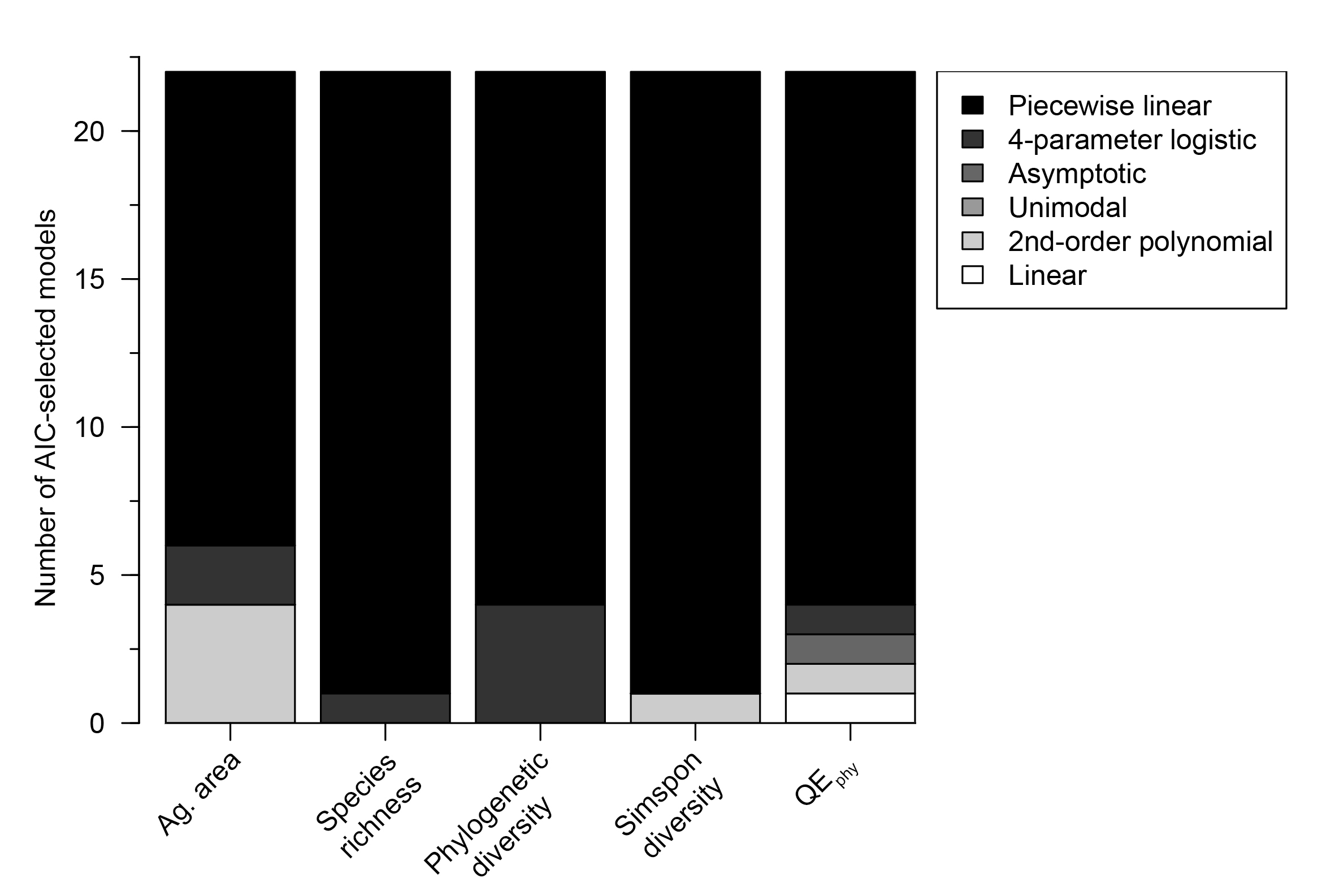


**Fig G. Number of AIC-selected models describing changes in agricultural area and four metrics of taxonomic and phylogenetic diversity through time across 22 regions.** Models are detailed in Equations 2-7 in the main text. AIC scores are presented in S1 File Table B, and final parameters for piecewise model fits are presented in S1 File Table C.


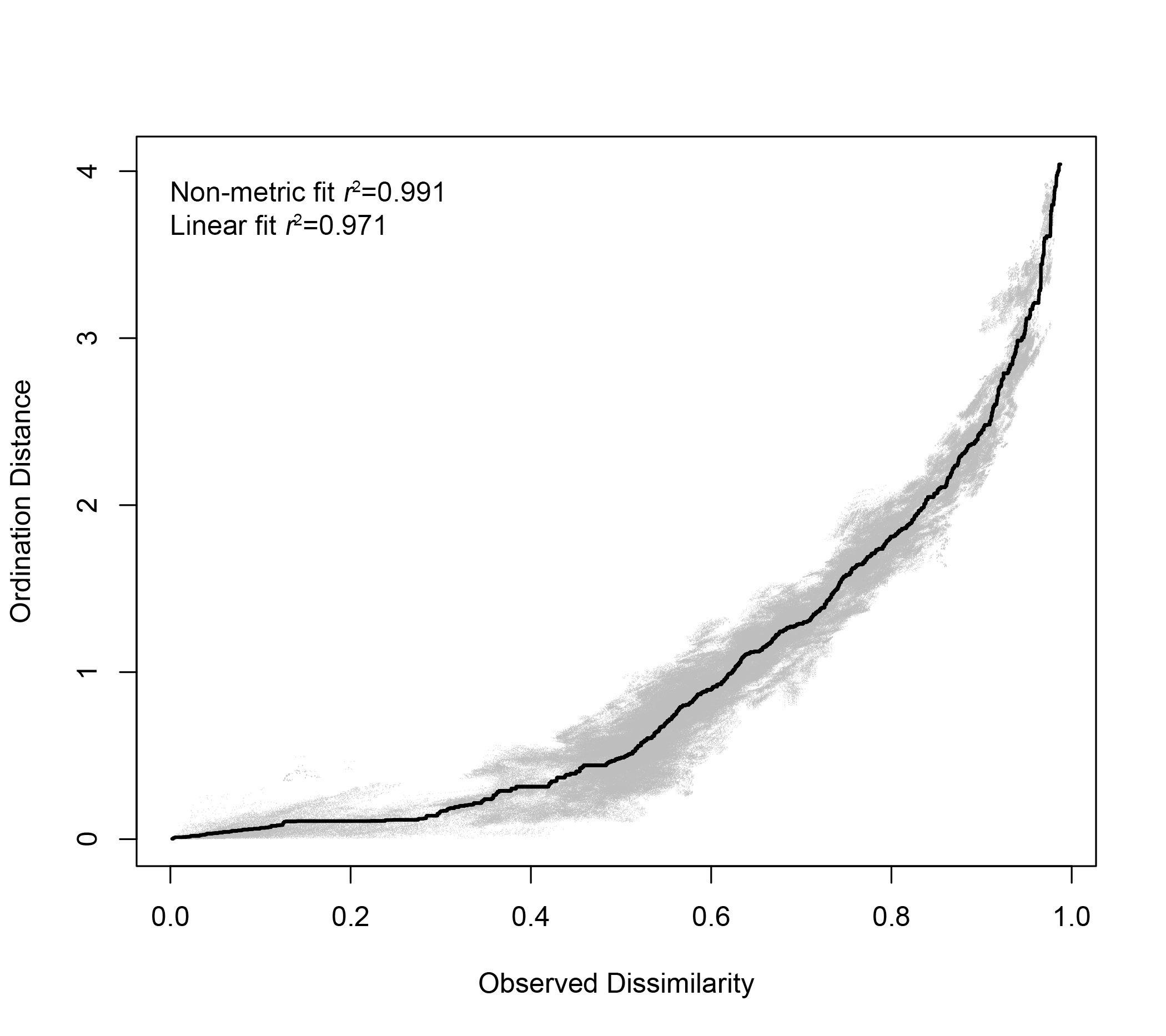


Fig H. Stress plot for abundance-weighted non-metric multidimensional scaling (NMDS) of region-by-year crop data. Pairwise distances were calculated as Bray-Curtis dissimilarity indices (see Equation 2-5 in main text), which were first subjected to a Wisconsin double standardization. Overall stress for the NMDS ordination was 0.094.

**Supplementary information references**

1. Webb CO, Ackerly DD, McPeek MA, Donoghue MJ. Phylogenies and community ecology. Annu Rev Ecol Syst. 2002;33:475-505. doi: 10.1146/annurev.ecolysis.33.010802.150448. PubMed PMID: WOS:000180007000018.

2. Li S-p, Cadotte MW, Meiners SJ, Hua Z-s, Jiang L, Shu W-s. Species colonisation, not competitive exclusion, drives community overdispersion over long-term succession. Ecol Lett. 2015;18:964–73.
